# Understanding metabolic alterations and heterogeneity in cancer progression through validated immunodetection of key molecular components: a case of carbonic anhydrase IX

**DOI:** 10.1007/s10555-021-10011-5

**Published:** 2022-01-26

**Authors:** Martina Takacova, Ivana Kajanova, Maria Kolarcikova, Jana Lapinova, Miriam Zatovicova, Silvia Pastorekova

**Affiliations:** Institute of Virology, Department of Tumor Biology, Biomedical Research Center of the Slovak Academy of Sciences, Dubravska cesta 9, 845 05 Bratislava, Slovak Republic

**Keywords:** Carbonic anhydrase IX, Metabolism, Heterogeneity, Hypoxia, Acidosis, Antibody validation

## Abstract

Cancer metabolic heterogeneity develops in response to both intrinsic factors (mutations leading to activation of oncogenic pathways) and extrinsic factors (physiological and molecular signals from the extracellular milieu). Here we review causes and consequences of metabolic alterations in cancer cells with focus on hypoxia and acidosis, and with particular attention to carbonic anhydrase IX (CA IX). CA IX is a cancer-associated enzyme induced and activated by hypoxia in a broad range of tumor types, where it participates in pH regulation as well as in molecular mechanisms supporting cancer cells’ invasion and metastasis. CA IX catalyzes reversible conversion of carbon dioxide to bicarbonate ion plus proton and cooperates with a spectrum of molecules transporting ions or metabolites across the plasma membrane. Thereby CA IX contributes to extracellular acidosis as well as to buffering intracellular pH, which is essential for cell survival, metabolic performance, and proliferation of cancer cells. Since CA IX expression pattern reflects gradients of oxygen, pH, and other intratumoral factors, we use it as a paradigm to discuss an impact of antibody quality and research material on investigating metabolic reprogramming of tumor tissue. Based on the validation, we propose the most reliable CA IX-specific antibodies and suggest conditions for faithful immunohistochemical analysis of molecules contributing to heterogeneity in cancer progression.

**Supplementary Information:**

The online version contains supplementary material available at 10.1007/s10555-021-10011-5.

## Introduction

During cancer progression, tumor cells are exposed to multiple physiological constraints present in the growing tumor tissue. These constraints dynamically change in spatial and temporal manner, generating selection pressures and adaptive responses leading to expansion of cancer cells that are able to survive and sustain proliferation. Selected cancer cell subpopulations exhibit phenotypic plasticity and invasive/pro-metastatic behavior, and contribute to complex tissue architecture with various physiological gradients. One of key adaptations to fluctuating supplies and demands for survival and proliferation of cancer cells is their metabolic reprogramming.

## Causes and consequences of metabolic alterations and heterogeneity

### Oncogenic activation

Initial tumor growth is associated with mutations that trigger deregulated oncogenic signaling. Activated oncoproteins and/or inactivated tumor suppressor proteins promote abnormal cell proliferation not only by activating positive regulators and counteracting negative regulators of the cell cycle, but also by redirecting cellular metabolism from oxidative phosphorylation to glycolysis [1–3]. They do so through signal transduction pathways leading to enhanced transcription, translation, and/or catalytic activation of the glycolytic enzymes (including glucose transporter GLUT, lactate dehydrogenase LDH, pyruvate dehydrogenase kinase PDK, and monocarboxylate transporter MCT) as explained in more detail elsewhere. Classical examples of this phenomenon include activated MYC and RAS and inactivated p53, von Hippel-Lindau (VHL), and phosphatase and tensin homolog (PTEN), but also many other regulatory molecules [4, 5]. In addition, activating mutations can occur directly in the components of the metabolic pathways, such as isocitrate dehydrogenase (IDH), fumarate hydratase (FH), and/or succinate dehydrogenase (SDH) [6–8]. Albeit glycolysis is not as efficient as respiration in energy production, it allows for synthesis of cellular biomass that is particularly important for uncontrolled proliferation of cancer cells [1, 2]. Yet, most cancer cells retain functional mitochondria that are essential for lipid synthesis and protein acetylation [6].

### Stresses in tumor microenvironment

Proliferative advantage of cancer cells enables *in situ* expansion of tumor lesion until the demands for nutrients and oxygen exceed their supply from the nearest functional blood vessel [9–11]. Long-lasting lack of oxygen (i.e., anoxia) is incompatible with the survival of cells and generates necrotic regions that are histological surrogates of poor cancer prognosis. Lowered oxygenation (i.e., hypoxia) reinforces the shift of cancer cells to glycolytic metabolism and induces additional phenotypic changes, such as angiogenesis generating aberrant tumor vasculature, cell–cell deadhesion linked with migration-invasion, and genomic instability facilitating accumulation of mutations [12]. Mechanisms behind these phenotypic changes include both transcriptional and translational reprogramming governed principally by the hypoxia-inducible transcription factors (HIFs) and by the unfolded protein response (UPR)-driven pathways, as reviewed in [13–16].

Importantly, these adaptations are not uniform. They vary depending on many circumstances including cancer type/origin, position of cells in the diffusion gradients, and time of their exposure to the hypoxic stress [11, 17]. Thus, metabolic preferences of the peri-necrotic cancer cells chronically suffering from severe hypoxia considerably differ from the preferences of the moderately hypoxic cells as well as from the oxygenated cells located proximally to the vasculature [18].

In a simplified interpretation, cells in the region of severe hypoxia are highly dependent on glycolysis, while the moderately hypoxic cells can utilize tricarboxylic acid (TCA) cycle and oxidative phosphorylation system (OXPHOS) by consuming lactate exported from the highly glycolytic cells, a phenomenon known as metabolic symbiosis [19, 20]. Normoxic cells exploit glycolysis in the presence of oxygen (so-called Warburg effect) along with TCA/OXPHOS, driven by various fuels available in the tumor microenvironment (TME), including amino acids and lipids [11, 17].

Oncogenic metabolism produces an excess of acidic metabolic products, such as lactate, protons, and carbon dioxide. Although these metabolites are primarily generated intracellularly, machinery protecting cancer cells from an intracellular acidosis extrudes them to the pericellular space either by the active export mechanisms or by altering/reversing their gradients across the plasma membrane [21]. Consequently, these acidic products accumulate in TME (especially when distant from the blood flow) and generate an extracellular acidosis, which has a significant impact on metabolic performance as well as on additional phenotypic characteristics of tumor cells contributing to their pro-metastatic behavior and to cancer progression [18, 22]. Acidosis was shown to directly affect protonation states of important regulatory proteins with pH-sensitive amino acids, as exemplified by p53, sodium-proton exchanger 1 (NHE1), epidermal growth factor receptor (EGFR), ß-catenin, etc., thereby causing structural changes that results in their altered functions [23]. Moreover, intracellular acidosis leads to increased accumulation of reactive oxygen species (ROS) and enhanced ferroptosis, a non-apoptotic form of iron-dependent cell death [24, 25]. Acidic pH is also linked with immune cells’ anergy and resistance to anticancer drugs [26].

Metabolic heterogeneity is also developing along the cancer progression stages. Invasive and circulating cancer cells depend on the acquisition of an anchorage-independence to avoid anoikis and gain metabolic phenotype overcoming excessive ROS production and bioenergetic crisis caused by the loss of attachment signals. These non-adherent cells require high amounts of ATP for survival (and not the biomass for proliferation), thus relying more on mitochondrial metabolism during their detachment, invasion, and flow within the blood stream or in lymphatics [27–29].

### Tumor-stroma cross-talk

Diversity of responses to physiological stresses in TME by cancer cell subpopulations is not the only determinants of the metabolic heterogeneity in tumors. This phenomenon gains complexity through the cross-talk of cancer cells with the cells in stroma. Stroma is a connective component of the tumor tissue showing highly variable extent and composition depending on the organ of residence, tumor type, and cancer stage. Different stromal cells communicate with cancer cells via multiple signaling entities including growth factors, exosomes, and micro-/nanotubular structures, but also via metabolites [17, 30, 31]. Cancer and stromal cells can live in a metabolic symbiosis not only by releasing/consuming lactate, but also by venting of cancer cells-produced CO_2_ via gap junctions of stromal cells and potentially through additional mechanisms that still remain to be elucidated [32, 33].

### Links between pH control and metabolism

Successful adaptation of tumor cells to oncogenic metabolism and/or hypoxia strongly depends on activation of the pH control machinery [18, 34]. Its prime role is to maintain a slightly alkaline intracellular pH (pHi ~ 7.2 to 7.7) that is required for the effective biosynthesis, ATP production, and signaling [35]. Major components of this pH control machinery can be induced by hypoxia and/or are pH-sensitive molecules, including sodium-proton exchangers (e.g., NHE1), anion exchangers (e.g., AE2), sodium-bicarbonate transporters (e.g., NBCe1), monocarboxylate transporters (MCT1 and MCT4), and related molecules. Moreover, MCTs can also affect the glycolytic flux by mediating co-extrusion of lactate and protons and therefore represent one of the intersecting points of metabolism and pH control [20, 22, 36].

Besides extrusion of lactate and protons, pHi control also involves removal of the intracellular CO_2_ by diffusion to extracellular space as well as the import of bicarbonate ions generated by the hydration of pericellular CO_2_ to cytoplasm, leaving protons from the same reaction outside the cells. These processes lead to extracellular acidosis that often persists in tumor microenvironment because the acidic metabolic products cannot be effectively removed by the aberrant tumor vasculature. Extracellular acidosis can activate proteolytic enzymes that degrade extracellular matrix and facilitate invasion and metastasis. Moreover, cancer cells with activated pH-regulating machinery can resist toxic effects of extracellular acidosis generated by oncogenic metabolism and even benefit from an acidosis-supported acquisition of aggressive tumor phenotypes. Thus, cancer cells gain selective advantage against surrounding normal cells that cannot adapt [37].

Acidosis strongly influences tumor metabolic preferences, reducing glycolysis while promoting mitochondrial activity. It supports progression-related processes such as angiogenesis, invasion, and metastasis and is linked to cellular phenomena including aneuploidy and mutation rate, autophagy and survival, cell migration, and release of exosomes [18]. Moreover, acidosis is enriched at tumor-stroma interfaces (in addition to regions of chronic hypoxia) and cells within the acidic front are invasive and proliferative [33, 38]. From the clinical point of view, intratumoral acidosis is associated with resistance to chemo-, radio-, and immuno-therapies.

## Carbonic anhydrase IX and its role in tumor metabolism

### CA IX essentials

Carbonic anhydrase IX (CA IX) has been initially cloned as a cancer-associated transmembrane enzyme with an active site facing the extracellular space and catalyzing the reversible conversion of carbon dioxide to bicarbonate ion and proton [39, 40]. CA IX shows very high catalytic activity, comparable to the CA II isoform that belongs to the most active enzymes in general. Interestingly, CA IX activity culminates at acidic pH around 6.5, typical for tumor microenvironment [41, 42]. Moreover, CA IX is strongly responsive to hypoxia both at the level of expression by the HIF-mediated transcriptional activation [43], and at the level of catalytic activity by the protein kinase A (PKA)-mediated phosphorylation [44]. In addition, available experimental data show that the expression of CA IX can be induced under normoxic conditions in connection with oncogenic activation of the RAS, SRC, or RET pathway, and upon treatment with glucose, lactate, and glutamine (particularly in combination with serum growth factors) that are important substrates of tumor metabolism [45–49].

**Fig. 1:**
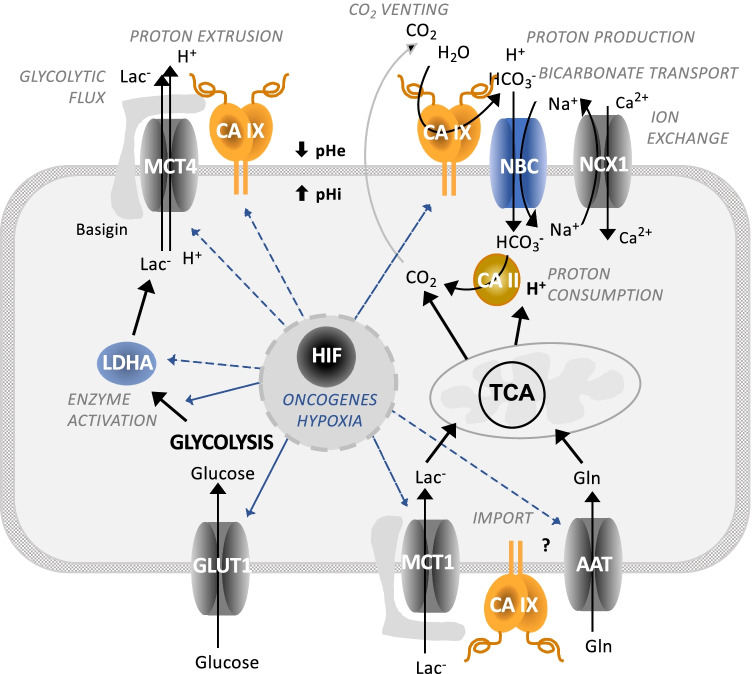
Schematic view of the CA IX position in molecular pathways driving metabolic reprogramming of cancer cells. Oncogenic activation and hypoxia drive metabolic reprogramming in part via HIF-mediated induction and/or activation of certain glycolytic enzymes (LDHA) and transporters of glucose (GLUT), lactate (MCT), and amino acids (AAT). This allows cancer cells to generate energy and biomass for survival and proliferation. At the same time, HIF induces key components of pH regulating machinery, including ion transporters (NBC and NCX) as well as carbonic anhydrase IX (CA IX), in order to protect cancer cells from intracellular acidosis generated by the oncogenic metabolism. CA IX cooperates with these molecules via its extracellular domains either in a catalytic or in a non-catalytic manner thereby regulating pH and supporting metabolic adaptations of cancer cells

CA IX is an important constituent of pH regulation in tumor cells via contribution to intracellular neutralization/alkalization and extracellular acidification [50–52]. Indeed, CA IX cooperates with a number of genuine pH regulators mentioned above, including NBCe1 and NBCn1, MCT1 and MCT4, NHE1, AE2, and NCX1 (Fig. 1) [53–59]. These cooperative activities are mainly dependent on the enzyme active site in the CA domain localized in the central part of the CA IX ectodomain near its transmembrane region, as described in [53–55] and depicted below. However, the N-terminal part (so-called PG region) protruding to the extracellular space participates in the non-catalytic pH regulation, as an antenna enhancing export of protons coupled with export of lactate ions via MCTs [56–58]. In fact, CA IX acts as an extracellular pH–stat, maintaining an acidic tumor extracellular pH that is tolerated by cancer cells and supports their pro-metastatic behavior [52]. CA IX-mediated extracellular acidosis is also associated with decreased immune activity in the tumors of patients with a broad spectrum of solid malignancies [60]. At the same time, CA IX stabilizes intracellular pH that is conducive to survival and proliferation [6, 51]. Recently, Chafe et al. have demonstrated that the role of CA IX in maintaining an alkaline intracellular pH is critical for suppression of ferroptosis [25].

The effects of CA IX on tumor phenotype can be blunted either by the silencing/suppression of the CA IX expression, or by the small molecule inhibitors of the CA IX catalytic activity, or by the CA IX-specific antibodies as documented in a number of published studies and reviews [61–68]. Moreover, the knowledge obtained in pre-clinical studies of CA IX inhibitors and/or antibodies has opened the window for novel anticancer strategies, some of which have been translated into the clinical trials (reviewed in [69] and listed in https://clinicaltrials.gov].

### CA IX and metabolic reprogramming

Since hypoxia and acidosis are intimately coupled with expression and activities of enzymes involved in oncogenic metabolism, it is not surprising that CA IX can play a role in metabolic pathways, echoing intratumoral oxygen and pH gradients in metabolic heterogeneity of tumor tissue. CA IX expression and/or activity appears to be required for accelerated lactate efflux via MCTs [57, 58], for full expression and activity of PDK1, a gate-keeping enzyme to the TCA cycle in mitochondria [70, 71], for full expression and activity of a key glycolytic enzyme LDHA [72], and ultimately for maximizing glycolytic flux and facilitating cell proliferation in hypoxic/acidic TME [73]. This conclusion is in line with the fact that glycolysis both generates and senses pH changes caused by the formation and accumulation of acidic metabolites [74, 75].

CA IX also plays a role in molecular mechanisms mediating cell adhesion-migration-invasion [76, 77]. On one hand, CA IX can affect the assembly and maturation of focal adhesion contacts during cell attachment and spreading on solid support [78, 79], and on the other one, it can destabilize E cadherin-mediated intercellular contacts [80] and facilitate migration/invasion. CA IX re-localizes to cellular leading-edge structures, namely filopodia during migration and invadopodia during invasion, where it contributes to pH regulation at both sides of the plasma membrane [54, 81]. It operates via coordinated regulation of an interactome composed of bicarbonate transporters and amino acid transporters as well as cortactin, integrins, and metalloproteinases [81–83]. This also implies possible involvement of CA IX in metabolic processes that supply energy both to (1) formation and growth of these highly dynamic subcellular structures potentially via podosome-confined glycolysis, as suggested by Stock and Schwab [84], and (2) movement and protrusion of individual cells or cell clusters via enhanced respiration, mitochondrial biogenesis, and reduced lipogenesis [85, 86].

### CA IX as a surrogate marker of hypoxia, acidosis, and glycolytic metabolism

CA IX is mostly viewed as a biomarker of hypoxia and/or acidosis. It is expressed in many tumor types and shows highly heterogeneous expression pattern, as reviewed in [68]. It is usually localized in broader peri-necrotic zones involving both highly and moderately hypoxic cells that are viable and possess a strong metastatic potential. Because of the responsiveness to both severe and moderate hypoxia, CA IX distribution only partly overlaps with that of HIF-1α and of other hypoxia-regulated proteins induced at different hypoxic thresholds [43, 87, 88]. Occasionally, CA IX distribution is diffused, presumably as a sign of oncogene activation or inactivating mutation of tumor suppressor. CA IX can be also found in HIF-1α negative areas possibly because reoxygenation leads to instantaneous degradation of the HIF-1α but not of the highly stable CA IX. In accord with the CA IX role in pH regulation, its expression is increased at the interface between tumor and stroma, in the acidic front containing invasive and proliferative cells that rely on glycolysis [9, 38].

**Fig. 2:**
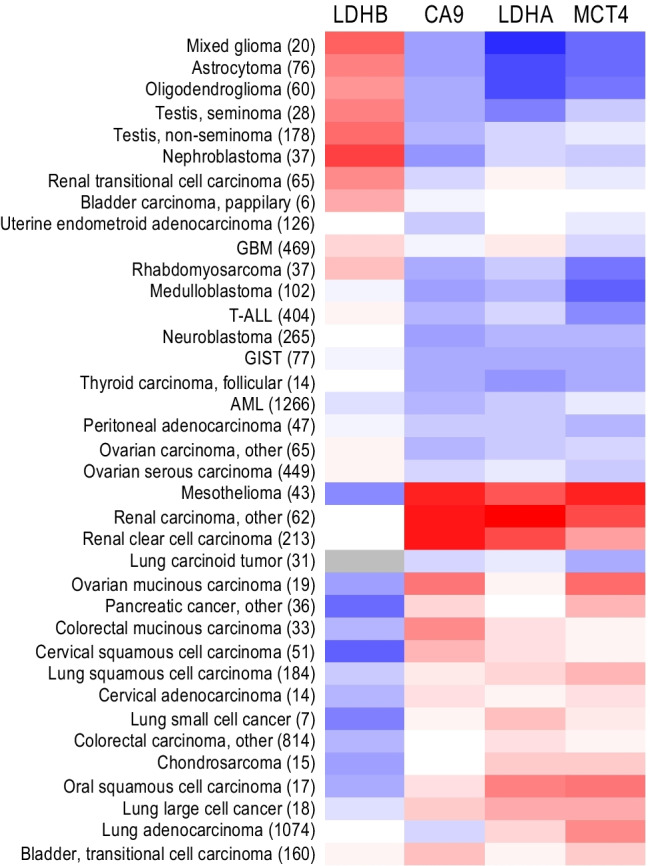
A heatmap visualizing differential expression of genes coding for the CA IX (CA9) and for the glycolytic enzymes LDHA (supporting glycolysis), LDHB (supporting TCA cycle), and MCT4 (extruding lactate ions and protons) in various tumor samples (the number of samples is indicated in brackets). The color scale ranges from blue (lowest mean expression) through white (average mean expression) to red (highest mean expression). Data were analyzed and extracted through IST (in silico transcriptomics) online (MediSapiens; https://medisapiens.com/), the largest fully integrated and annotated human gene expression data source

In light of the data connecting CA IX to glycolytic metabolism, CA IX can be viewed as a surrogate indicator of glycolytic metabolic phenotype. Indeed, data from the literature show that in patients’ specimens of tumors derived from diverse tissue types, CA IX is often correlated, co-expressed, and/or spatially overlapped with the traditional biomarkers of the glycolytic metabolism (GLUT1, MCTs, LDH) and glucose consumption rate (^18^FDG). For example, significant overall correlation and co-localization of CA IX with GLUT1, MCT4, and MCT1 was demonstrated by immunohistochemistry (IHC) in advanced head and neck carcinomas [89]. Considerable spatial overlap between CA IX and GLUT1 was found in areas of diffusion-limited hypoxia in glioblastomas and astrocytomas [90]. CA IX correlation with GLUT1 was also found in papillary renal cell carcinomas [91], in bladder cancer [92], and in cervical carcinomas [93]. In addition, higher expression of CA IX was linked with the stronger ^18^FDG uptake in non-small cell lung cancer [94]. CA IX expression was also correlated with LDH5 expression in gastrointestinal adenocarcinomas [95]. Interestingly, analysis of transcriptional profiles of different tumor types fully supports these links (Fig. 2). Altogether, these data reinforce the existence of *in vivo* link between CA IX and glycolysis.

## Challenges for characterizing metabolic heterogeneity in tumor tissues

Current research of cancer metabolism and metabolic heterogeneity in cancer progression focuses primarily on technologically advanced methods of metabolomics, as reviewed by [96]. However, identification of underlying molecular mechanisms still depends on approaches of molecular/cell biology and cancer physiology. Due to a complex nature of this topic, it is extremely important to employ validated research tools and correct methodical approaches as well as appropriate research material.

The need for antibody validation is particularly evident for molecules that are functionally involved in metabolic pathways, irrespective of whether they are detected in cellular extracts, cultured cells, or in tumor tissues [97]. Once the antibody does not fulfill strict specificity and quality requirements, it can provide false data leading to inaccurate interpretation of results. This can eventually obscure the recognition of a real biological role and/or clinical value of the studied molecule. In addition, such situation can result in impairment of data reproducibility and failure of therapeutic targeting strategies, as witnessed in nowadays’ science and clinical R&D. There are also additional challenges including inappropriate tissue material and data presentation that have to be addressed in order to improve the way towards unraveling tumor heterogeneity.

### Quality of antibodies

Looking at the investigations of CA IX as a molecule that contributes to tumor heterogeneity and metabolic reprogramming through response to hypoxia and acidosis, it is evident that researchers use diverse antibodies from various sources, often without proper characterization, just relying on the recommendations in datasheet. To date, more than 1,310 (from 45 providers) and 959 anti-CA IX antibodies are listed in Antibodypedia [98] and CiteAb [99], respectively. Many of these antibodies are routinely used in basic research for detection and quantification of CA IX, as well as for determination of its distribution and interactions within cells. A subgroup of antibodies is also used in clinical studies to reveal the CA IX expression in tissues and its prognostic, diagnostic, and therapeutic potential. However, not all of these antibodies can comply with high standards of performance and reliability.

**Fig. 3:**
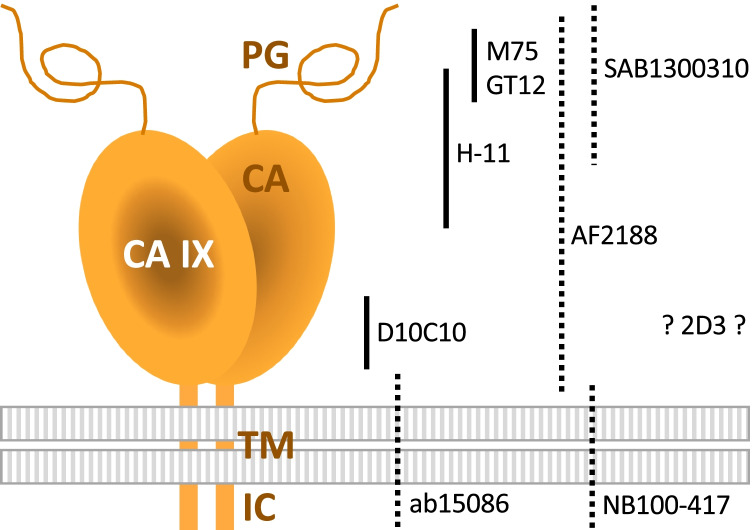
Schematic illustration of binding sites of M75 and eight validated antibodies. The positions of antibody binding regions are shown on the schematic model of the CA IX protein comprising a proteoglycan-like (PG) region, carbonic anhydrase (CA) domain, transmembrane (TM) anchor, and intracytoplasmic (IC) tail. Antibody arrangement reflects the information available in the datasheets. Monoclonal and polyclonal type of antibody is depicted using full and dashed line, respectively. No information regarding the immunogen used for the generation of 2D3 monoclonal antibody is available in its datasheet. According to “Ten basic rules of antibody validation” [101], all selected antibodies are correctly described by providers and, with exception of 2D3, have a defined immunogen

**Fig. 4:**
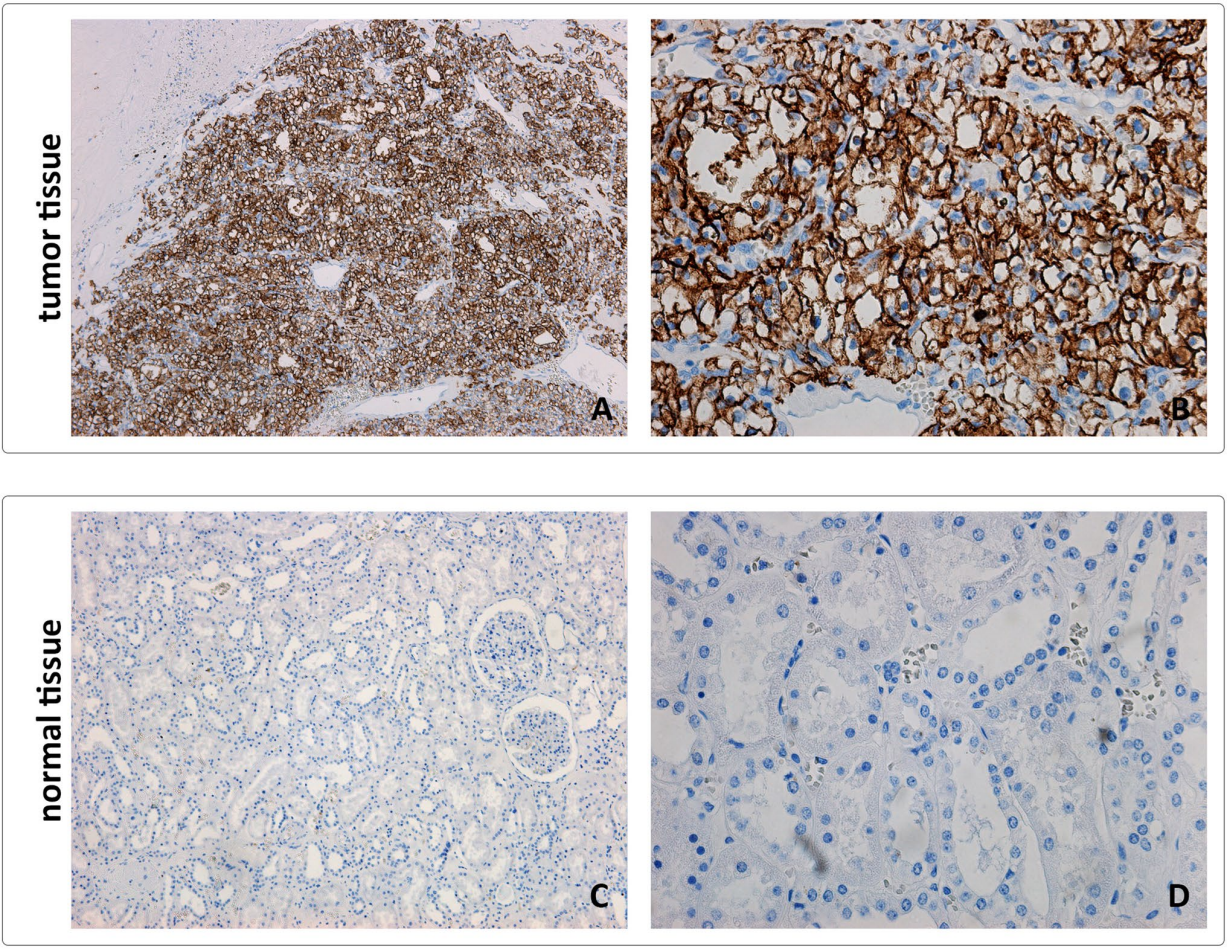
Representative images from tumor (CCRCC) and normal kidney tissue stained using M75 reference antibody. Tissue sections were directly incubated with M75 monoclonal antibody for 1 h at RT. Following the incubation with anti-mouse secondary antibody, positive reaction was visualized using DAB as a chromogen. Sections were counterstained with Mayer hematoxylin. **A**, **C** Original magnification × 100. **B**, **D** Original magnification × 400

To clarify this situation, eight commercially available antibodies that are most frequently cited in the numerous clinical studies of CA IX were subjected to a comprehensive validation in our laboratory. Overall reactivity of the antibodies was compared with the providers’ recommendations in the datasheets. Table 1 summarizes an overview of the antibody validation results obtained in five applications, namely WB, ELISA, FACS, IF/ICC, and IHC (see the original validation data in the Supplementary information). Antibody binding regions on the CA IX molecule are depicted in Fig. 3. The antibodies were selected predominantly on the basis of meta-analysis performed by van Kuijk and colleagues [100]. There, the data from 147 clinical studies encompassing more than 24,000 cancer patients were evaluated with respect to CA IX expression assessed by IHC in relationship to several endpoints, including overall survival (OS), disease-free survival (DFS), and progression-free survival (PFS). Meta-analysis confirmed the correlation of high CA IX expression to disease progression, locoregional failure, and development of metastasis, independently of tumor type or site. Since most of the included clinical studies (46%) employed M75 monoclonal antibody, it was used as a reference antibody. Moreover, its CA IX specificity and excellent performance was proven in many other research papers from a number of laboratories. Representative IHC staining with M75 antibody is shown in Fig. 4.

**Table 1:**
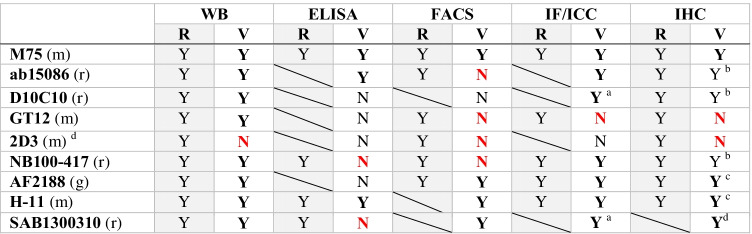
Characteristics of antibodies demonstrated by validation compared to recommendations of providers

^a^Only in cells with high ectopic expression

^b^Only after demasking

^c^High background

^d^Clone with the same characteristics is available also as NBP1-51691 and ab107257

^d^Faint staining signal

*R*, recommended by the provider; *V*, validated in our laboratory; *Y*, yes; *N*, no; *m*, mouse; *r*, rabbit; *g*, goat; red color signifies a disagreement with the provider’s recommendation

Because IHC is (except metabolomics) a key approach in determining tissue expression of regulators/executors of metabolic reprogramming in tumors and in elucidating their clinical value as potential biomarkers and therapy targets, our validation was focused primarily on this application. Specificity of anti-CA IX antibodies in IHC was examined using serial tissue sections of the clear cell renal cell carcinoma (CCRCC) with proven high expression of CA IX compared to the CA IX-negative normal kidney tissue (Supplementary information). In addition to the M75 reference, additional 3 commercial antibodies were found more or less suitable for IHC, considering specific methodical adjustments, such as requirement for antigen retrieval (citrate or EDTA) as well as duration and temperature of staining (1 h at RT versus O/N at 4 °C). Assessment of these methodical details for each particular antibody and their precise description in related publication is extremely important for the reliability and reproducibility of data.

In brief, the validation performed in our laboratory revealed that monoclonal D10C10 and polyclonal antibodies ab15086 and NB100-417 are reliable alternatives to monoclonal antibody M75, while the antibodies H-11 and AF2188 suffer from non-specific reactivity and high background that may potentially lead to false positivity. The antibodies GT12 and 2D3 are not suitable for the detection of CA IX by IHC.

### Identification of antibodies and data presentation

Almost 30% of the IHC studies involved in the meta-analysis by van Kuijk et al. (2016) [100] contain insufficient description of the used anti-CA IX antibody, missing its unambiguous identification and just mentioning the provider (if at all). In some cases, authors declare an incorrect source of the antibody (see Table 2). Since some providers offer a number of different antibodies to CA IX, this introduces a lot of confusion and questions the data interpretation as well as their reproducibility. However, this drawback is not limited to CA IX—it is a generally occurring and persisting problem in the entire biomedical research, since the antibody cannot be recognized in more than a half of all respective publications (as reviewed by [101]).

**Table 2:** Evaluation of selected clinical studies (first author, year) with respect to their reproducibility potential. The studies were clustered according to organ of tumor origin and subjected to evaluation of the primary antibody quality based on the results of our current validation (antibody), relevance of tissue specimens for investigation of tumor heterogeneity (tissue), and methodology judged according to the available data on antigen retrieval, staining kits, and positive/negative control (IHC staining). The “reproducibility index” (RI) was calculated as a sum of stars awarded to each variable (Antibody, Tissue, IHC staining) with the maximum of 7 points per study. Three stars were used when a sufficient description (clone name, source, dilution) of primary anti-CA IX antibody was mentioned within a particular study. Tissue sample was evaluated using either T/TMA≧3 (for whole tissue or TMA ≧ 3 cores, 1 star) or B/TMA < 3 (for biopsy or TMA < 3 cores, no star). IHC staining was evaluated regarding the available data about antigen retrieval, staining kit, and positive/negative control (maximum 3 stars). Hazard ratio (HR) with corresponding 95% CI as well as Newcastle–Ottawa score (NOS) quality assessment was adopted from van Kuijk et al., Front Oncol 2016 [100]

Brain						
Study	Antibody	Tissue	IHC staining	RI	HR (95% CI)/endpoint	NOS
Dungwa, 2012 [102]	M75 ✶ ✶	T/TMA≧3 ✶	✶ ✶	**5**	1.71 (0.80–3.65)/OS 1.73 (0.83–3.59)/PFS	7
Korkolopoulou, 2007 [103]	M75 ✶ ✶^R^	T/TMA≧3 ✶	✶ ✶ ✶	6*	4.04 (2.38–6.85)/OS	6
Ameis, 2015 [104]	M75 ✶ ✶ ✶	T/TMA≧3 ✶	✶ ✶	**6**	15.7 (2.04–121.1)/OS	5
Jarvela, 2008 [105]	M75 ✶ ✶ ✶	B/TMA < 3	✶ ✶	**5**	2.98 (1.67–5.30)/OS	5
Nordfors, 2010 [106]	M75 ✶ ✶ ✶	B/TMA < 3	✶ ✶	**5**	3.96 (1.20–13.0)/OS	4
Haapasalo, 2006 [107]	M75 ✶ ✶ ✶	B/TMA < 3	✶ ✶ ✶	**6**	1.40 (1.01–1.94)/0S	3
Erpolat, 2013 [108]	Abcam ✶ ✶	T/TMA≧3 ✶	✶ ✶ ✶	**6**	2.34 (1.47–3.71)/OS	5
Proescholdt, 2012 [109]	Novus ✶ ✶	B/TMA < 3	^R^	2*	3.67 (2.03–6.61)/OS	6
Yoo, 2010 [110]	Novus ✶ ✶	T/TMA≧3 ✶	✶ ✶	**5**	2.27 (1.29–4.00)/OS	4
Abraham, 2012 [111]	†Santa Cruz ✶ ✶	T/TMA≧3 ✶	✶ ✶ ✶	**6**	1.59 (0.35–7.15)/OS 1.65 (0.50–5.48)/PFS	5
Abraham, 2012 [111]	†Santa Cruz ✶ ✶	T/TMA≧3 ✶	✶ ✶ ✶	**6**	0.19 (0.02–1.61)/OS 0.47 (0.06–3.69)/PFS	5
Jensen, 2012 [112]	†Santa Cruz ✶ ✶	T/TMA≧3 ✶	✶ ✶ ✶	**6**	1.10 (0.62–1.95)/OS 1.31 (0.64–2.66)/PFS	4
Sooman, 2015 [113]	Strategic Diagnostics ✶ ✶ ✶	B/TMA < 3	^R^	3*	3.35 (1.55–7.22)/OS	4
Flynn, 2008 [114]	**?**	T/TMA≧3 ✶	✶ ✶ ✶	**4**	1.19 (0.70–2.03)/OS	4
Preuser, 2005 [115]	**?**	T/TMA≧3 ✶	✶ ✶	**3**	Inadequate data/OS	-
**Pancreas**						
Study	Antibody	Tissue	IHC staining	RI	HR (95% CI)/endpoint	NOS
Couvelard, 2005 [116]	M75 ✶ ^a^	T/TMA≧3 ✶	✶ ✶ ✶	**5**	35.3 (10.3–121)/OS	7
Couvelard, 2005 [117]	M75 ✶ ^a^	T/TMA≧3 ✶	✶ ✶ ✶	**5**	1.86 (0.90–3.84)/OS	7
Chang, 2010 [118]	M75 ✶ ✶ ^R^	T/TMA≧3 ✶	✶ ✶	5*	0.99 (0.54–1.80)/OS	4
Hiraoka, 2010 [119]	M75 ✶ ✶ ✶	T/TMA≧3 ✶	✶ ✶ ✶	**7**	1.33 (0.97–1.84)/DFS 1.49 (1.07–2.07)/DSS	3
Li, 2016 [120]	Abcam ✶ ✶	T/TMA≧3 ✶	✶ ✶ ✶	**6**	2.24 (1.26–3.96)/OS	7
Schmitt, 2009 [121]	Abcam ✶ ✶ ✶	B/TMA < 3	✶ ✶ ✶	**6**	7.36 (3.11–17.4)/DFS	3
Yu, 2015 [122]	Epitomics ✶ ✶	T/TMA≧3 ✶	✶ ✶ ✶	**6**	1.07 (0.63–1.81)/OS	6
**Breast**						
Study	Antibody	Tissue	IHC staining	RI	HR (95% CI)/endpoint	NOS
Trastour, 2007 [123]	M75 ✶ ✶ ✶	T/TMA≧3 ✶	✶ ✶	**6**	Inadequate data/OS 2.57 (1.39–4.77)/DFS 2.70 (1.20–6.10)/MFS	7
Hussain, 2007 [124]	M75 ✶ ✶ ✶	T/TMA≧3 ✶	✶ ✶ ✶	**7**	2.63 (1.21–5.70)/OS	6
Betof, 2012 [125]	M75 ✶ ✶^a^	B/TMA < 3	✶ ✶ ✶	**5**	2.20 (1.10–4.41)/OS 1.88 (1.13–3.13)/PFS	5
Aomatsu, 2014 [126]	M75 ✶ ✶^a^	T/TMA≧3 ✶	✶ ✶	**5**	4.44 (1.80–10.9)/DFS	4
Lou, 2011 [127]	M75 ✶ ✶^R^	B/TMA < 3	✶^R^	3**	1.93 (1.65–2.26)/DFS 2.28 (1.89–2.73)/DSS 2.06 (1.74–2.43)/MFS	4
Tan, 2009 [128]	M75 ✶ ✶ ✶	B/TMA < 3	✶ ✶	**5**	5.02 (3.01–8.38)/OS 1.89 (1.19–3.00)/DFS	4
Brennan, 2006 [129]	M75 ✶ ✶^a^	B/TMA < 3	✶ ✶ ✶	**5**	1.91 (1.12–3.26)/OS 1.99 (1.30–3.05)/DFS 1.85 (1.19–2.87)/DSS	3
Generali, 2006 [130]	M75 ✶ ✶ ✶	B/TMA < 3	✶ ✶ ✶	**6**	1.93 (0.86–4.33)/OS 1.67 (0.89–3.14)/DFS	3
Kyndi, 2008 [131]	M75 ✶ ✶^R^	B/TMA < 3	✶ ✶ ✶	5*	1.30 (1.06–1.60)/OS 1.28 (0.82–2.01)/LC 1.29 (1.02–1.62)/DSS 1.23 (0.98–1.54)/MFS	3
Neumeister, 2012 [132]	M75 ✶ ✶ ✶	B/TMA < 3	✶ ✶ ✶	**6**	2.21 (1.20–4.09)/DSS	3
Neumeister, 2012 [132]	M75 ✶ ✶ ✶	B/TMA < 3	✶ ✶ ✶	**6**	1.36 (0.75–2.47)/DSS	3
Tomes, 2003 [88]	M75 ✶ ✶ ✶	T/TMA≧3 ✶	✶ ✶ ✶	**7**	0.78 (0.31–1.94)/OS	2
Doyen, 2014 [133]	M75 ✶ ✶ ✶	T/TMA≧3 ✶	✶ ✶	**6**	Inadequate data/MFS	-
Pinheiro, 2011 [134]	Abcam ✶ ✶ ✶	B/TMA < 3	✶ ✶ ✶	**6**	2.22 (0.79–6.20)/DFS	7
Garcia, 2007 [135]	Abcam ✶ ✶ ✶	B/TMA < 3	✶ ✶	**5**	1.55 (1.19–2.01)/OS	4
Bane, 2014 [136]	Abcam ✶ ✶	T/TMA≧3 ✶	✶	**4**	1.53 (0.84–2.77)/LC	4
Kim H, 2014 [137]	Abcam ✶ ✶	B/TMA < 3	✶ ✶ ✶	**5**	1.43 (0.80–2.56)/OS	4
Lancashire, 2010 [138]	Abcam ✶ ✶ ✶	B/TMA < 3 T/TMA≧3 ✶	✶ ✶ ✶	6*	Inadequate data/OS 2.19 (0.78–6.16)/DFS	3
Kornegoor, 2012 [139]	Abcam ✶ ✶	T/TMA≧3 ✶	✶ ✶ ✶	**6**	0.62 (0.19–2.11)/OS	3
Noh, 2014 [140]	Abcam ✶ ✶	B/TMA < 3	✶ ✶ ✶	**5**	Inadequate data/OS inadequate data/DFS	-
Currie, 2013 [141]	Novus ✶ ✶	T/TMA≧3 ✶	✶ ✶ ✶	**6**	1.05 (0.48–2.26)/OS 0.77 (0.39–1.55)/DFS	6
Deb, 2014 [142]	Novus ✶ ✶	B/TMA < 3	✶ ✶	**4**	2.20 (0.80–5.70)/OS	3
Beketic-Oreskovic, 2011 [143]	†Santa Cruz ✶ ✶	T/TMA≧3 ✶	✶ ✶ ✶	**6**	4.78 (2.15–10.6)/OS	7
Kaya, 2012 [144]	†Santa Cruz ✶ ✶ ✶	T/TMA≧3 ✶	✶ ✶ ✶	**7**	2.82 (1.71–4.64)/OS 0.84 (0.53–1.33)/DFS	5
**Head and neck**						
Study	Reproducibility index				HR (95% CI)/endpoint	NOS
	Antibody	Tissue	IHC staining	RI		
Silva, 2010 [145]	M75 ✶ ✶ ✶	B/TMA < 3	✶ ✶ ✶	**6**	5.21 (2.48–10.9)/LC 5.08 (2.53–10.2)/DSS	7
Hui, 2002 [87]	M75 ✶ ✶	B/TMA < 3	✶ ✶ ✶	**5**	1.39 (0.64–3.01)/OS 1.28 (0.65–2.52)/PFS	6
Koukourakis, 2001 [146]	M75 ✶ ✶ ✶	B/TMA < 3	✶ ✶	**5**	2.47 (1.23–4.98)/OS 3.05 (1.46–6.35)/LC	6
Koukourakis, 2006 [147]	M75 ✶ ✶	B/TMA < 3	✶ ✶	**4**	1.79 (1.21–2.64)/OS 1.84 (1.24–2.75)/LC	6
Bernstein, 2015 [148]	M75 ✶ ✶ ✶	B/TMA < 3	✶ ✶ ✶	**6**	1.67 (0.77–3.61)/LC 2.31 (1.04–5.12)DSS	5
Choi, 2008 [149]	M75 ✶ ✶ ✶	T/TMA≧3	✶ ✶	**6**	1.91 (0.77–4.71)/OS	5
De Schutter, 2005 [150]	M75 ✶ ✶ ✶	B/TMA < 3	✶ ✶ ✶	**6**	1.68 (0.94–2.99)/DFS 1.72 (0.94–3.14)/LC	5
Douglas, 2013 [151]	M75 ✶ ✶ ✶	B/TMA < 3	✶ ✶ ✶	**6**	1.76 (0.87–3.56)/LC	5
Heo, 2012 [152]	M75 ✶ ✶ ✶	T/TMA≧3 ✶	✶ ✶	**6**	4.95 (1.39–17.5)/DFS	5
Kim S, 2007 [153]	M75 ✶ ✶^a^	T/TMA≧3 ✶	✶ ✶ ✶	**6**	2.99 (1.39–6.45)/OS 1.76 (0.89–3.51)/DFS	5
Perez-Sayans, 2012 [154]	M75 ✶ ✶ ✶	B/TMA < 3	✶ ✶ ✶	**6**	1.36 (0.43–4.26)/OS	4
Wachters, 2013 [155]	M75 ✶ ✶ ✶	T/TMA≧3 ✶	✶ ✶	**6**	0.83 (0.31–2.22)/OS 0.34 (0.04–2.58)/LC	4
Eriksen, 2007 [156]	M75 ✶ ✶ ✶	T/TMA≧3 ✶	✶ ✶ ✶	**7**	1.10 (0.74–1.64)/LC	3
Le, 2005 [157]	M75 ✶ ✶^R^	B/TMA < 3	✶ ^R^	3**	1.73 (0.91–3.29)/OS 2.21 (1.11–4.39)/DFS	3
Nordsmark, 2007 [158]	M75 ✶ ✶^a^	T/TMA≧3 ✶	✶ ✶	**5**	1.27 (0.62–2.62)/LC	2
Winter, 2006 [159]	M75 ✶ ✶^R^	B/TMA < 3	✶ ✶ ✶	5*	Inadequate data/OS inadequate data/DFS inadequate data/DSS	-
Koukourakis, 2008 [160]	Abcam ✶ ✶	T/TMA≧3 ✶ B/TMA < 3	✶ ✶	4*	1.64 (0.62–4.32)/OS 4.41 (1.72–11.3)/LC	5
Kondo, 2011 [161]	Abcam ✶ ✶	T/TMA≧3 ✶	✶ ✶	**5**	3.36 (0.97–11.7)/OS	4
Brockton, 2011 [162]	Abcam ✶ ✶	T/TMA≧3 ✶	✶ ✶	**5**	1.20 (0.46–3.12)/OS	4
Brockton, 2012 [163]	Abcam ✶ ✶ ✶ M75 ✶ ✶^a^	T/TMA≧3 ✶	✶ ✶ ✶	**7**	2.04 (0.76–5.49)/DSS	3
Zheng, 2015 [164]	Cell Signaling ✶	B/TMA < 3	✶ ✶	**3**	4.27 (2.37–7.72)/OS	4
Sakata, 2008 [165]	Novus ✶	B/TMA < 3	^**R**^	1*	0.91 (0.32–2.61)/LC	7
Chen, 2014 [166]	Novus ✶ ✶	B/TMA < 3	✶ ✶	**4**	1.97 (1.02–3.81)/OS 1.96 (1.01–3.78)/LC 1.96 (1.02–3.76)/MFS 2.01 (1.05–3.86)/PFS	6
Hwa, 2015 [167]	Novus ✶ ✶	B/TMA < 3	✶ ✶	**4**	0.29 (0.05–1.77)/DSS	4
Kwon, 2015 [168]	Novus ✶ ✶	B/TMA < 3	✶ ✶	**4**	8.65 (1.01–74.1)/LC	4
Han, 2012 [169]	R&D Systems ✶ ✶ ✶	B/TMA < 3	✶	**4**	0.65 (0.12–3.67)/OS 0.50 (0.80–3.15)/DFS	6
Roh, 2008 [170]	R&D Systems ✶ ✶ ✶	B/TMA < 3	-	**3**	0.71 (0.23–2.22)/OS 1.77 (0.56–5.56)/DFS 1.20 (0.34–4.18)/LC	5
Roh, 2009 [171]	R&D Systems ✶ ✶ ✶	B/TMA < 3	-	**3**	1.09 (0.43–2.76)/LC 2.13 (0.74–6.13)/DSS	4
Yang, 2015 [172]	†Santa Cruz ✶ ✶	B/TMA < 3	✶ ✶	**4**	1.76 (1.07–2.87)/OS	7
Eckert, 2010 [173]	†Santa Cruz ✶ ✶ ✶	B/TMA < 3	✶	**4**	1.34 (0.65–2.76)/OS	5
Jonathan, 2006 [174]	**?**	B/TMA < 3	✶ ✶	**2**	0.27 (0.08–0.93)/LC 0.27 (0.09–0.83)/MFS	2
Rademakers, 2013 [175]	**?**	B/TMA < 3	✶ ✶	**2**	0.70 (0.50–1.10)/OS 0.50 (0.20–1.10)/LC 0.70 (0.40–1.50)/MFS	1

^R^Relevant information was supplied as a reference

^†^Antibody no longer available

^a^Incorrect reference to the origin or source of the M75 antibody

? no data available

Moreover, about 14% of papers included in the meta-analysis [100]do not display even the representative images of the IHC stained tissues (neither in the articles themselves, nor in their Supplementary data), thus precluding a visual inspection of the staining intensity and pattern.

### Tissue specimens

Deeper analysis of all CA IX-dedicated IHC studies summarized by van Kuijk and colleagues [100] revealed another problem. Whole tissue blocks were used in 40% of studies, the rest was performed using either biopsy or tissue microarray (TMA). Although there are several advantages favoring the utilization of TMAs in IHC, this approach can lead to loss of important information due to intratumoral heterogeneity, especially when only one TMA core is included in the staining. This is supported by the observation that only 4.7% of breast cancer TMA cores were found CA IX-positive compared to 18.1% of whole tissue blocks [138]. Another factor negatively impacting results of IHC lies in omitting perinecrotic regions from TMA construction. It is well-known that intratumoral hypoxia is reflected histologically by the presence of necrosis, which is considered a bad prognostic factor in cancer patients [176, 177]. Staining pattern of the hypoxia-induced CA IX is very heterogeneous, often confined to perinecrotic areas, and therefore, use of TMAs devoid of necrotic regions is not appropriate for the CA IX IHC analysis. The same is true for other molecules related to cancer metabolism, which is a phenomenon with inherent heterogeneity.

In case of TMA, the best way how to overcome the problem of tumor tissue heterogeneity is to analyze multiple cores. According to several studies, up to 98% consistency with the results from full-block sections can be achieved when at least three TMA cores are stained. Lower concordance is observed with only 1 or 2 cores (as reviewed in [178]). In meta-analysis presented by van Kuijk et al. (2016), 3 or more cores were used in 41.7% of TMA-based IHC studies, and less than three cores were used in 38.3% cases, whereas in the rest of the studies, tissue selection was not properly defined.

While insufficient identification and quality of antibodies clearly contribute to replication crisis in general, use of inappropriate tissue material particularly jeopardizes understanding of tumor heterogeneity including its metabolic aspects.

Deeper analysis of the 147 clinical studies mentioned in the meta-analysis by van Kuijk and colleagues revealed some general weak points frequently occurring in multiple clinical studies, e.g., insufficient description of antibody (clone name, source, dilution) and staining procedure (antigen retrieval, staining kit, positive/negative control), and tissue selection (whole tissue block vs TMA). As shown in Table 2, reevaluation of these studies with respect to their reliability and reproducibility using a “reproducibility index” (RI) based on inspection of the antibody properties and staining methodology showed remarkable discrepancies with an established Newcastle–Ottawa scale (NOS) (which was used by van Kuijk and colleagues) [100, 179]. The traditional NOS evaluation system puts emphasis on the scoring methodology, the cohort characteristics, and the disease outcome, while it ignores the abovementioned important aspects of IHC studies, such as precise description of antibody, type of tissue specimen, and details of staining procedure. This can significantly impact on the interpretation of data and ultimately affect their translation to clinical side.

## Conclusion

Understanding physiological and molecular mechanisms of cancer metabolic plasticity requires not only technologically advanced high-throughput metabolomic, proteomic, and genomic approaches, but also classical methods of molecular and cellular biology. The latter approaches have already uncovered spectrum of molecules and pathways driving metabolic reprogramming that facilitates survival and proliferation of cancer cells in the process of tumor tissue growth as well as during metastatic dissemination. These molecules include metabolic enzymes, transporters, and regulators that often display highly heterogeneous expression pattern reflecting dynamically changing selection-adaptation forces in tumor microenvironment. Using the example of CA IX, we provide a basic insight into the interplay of these molecules. Through the validation of CA IX-specific antibodies, we explain that well-characterized research tools/materials and sufficient technical details are important prerequisites for acquisition of reliable/reproducible data and for building of new knowledge translatable from bench to bedside.

## Supplementary Information

Below is the link to the electronic supplementary material.

Supplementary file1 (PDF 10594 KB)

## Abbreviations

AAT: Amino acid transporter
AE: Anion exchanger
CA IX: Carbonic anhydrase IX
CCRCC: Clear cell renal cell carcinoma
DSF: Disease-free survival
ECD: Extracellular domain
ELISA: Enzyme-linked immunosorbent assay
FACS: Fluorescence-activated cell sorting
FL: Full length
GLUT: Glucose transporter
HIF: Hypoxia-inducible factor
HRE: Hypoxia-responsive element
IC: Intracytoplasmic
IF: Immunofluorescence
IHC: Immunohistochemistry
LDH: Lactate dehydrogenase
MCT: Monocarboxylate transporter
NBC: Sodium bicarbonate cotransporter
NHE: Sodium-proton exchanger
OS: Overall survival
OXPHOS: Oxidative phosphorylation
PDK: Pyruvate dehydrogenase kinase
PFS: Progression-free survival
PG: Proteoglycan
RI: Reproducibility index
ROS: Reactive oxygen species
TCA: Tricarboxylic acid
TM: Transmembrane
TME: Tumor microenvironment
WB: Western blotting

## References

[1] Vander Heiden MG, Cantley LC, Thompson CB (2009). Understanding the Warburg effect: The metabolic requirements of cell proliferation. Science.

[2] Levine AJ, Puzio-Kuter AM (2010). The control of the metabolic switch in cancers by oncogenes and tumor suppressor genes. Science.

[3] Nagarajan A, Malvi P, Wajapeyee N (2016). Oncogene-directed alterations in cancer cell metabolism. Trends Cancer.

[4] Hsieh AL, Walton ZE, Altman BJ, Stine ZE, Dang CV (2015). MYC and metabolism on the path to cancer. Seminars in Cell & Developmental Biology.

[5] Mukhopadhyay S, Vander Heiden MG, McCormick F (2021). The metabolic landscape of RAS-driven cancers from biology to therapy. Nat Cancer.

[6] Schulze A, Harris AL (2012). How cancer metabolism is tuned for proliferation and vulnerable to disruption. Nature.

[7] Sciacovelli M, Frezza C (2016). Oncometabolites: Unconventional triggers of oncogenic signalling cascades. Free Radical Biology & Medicine.

[8] Schmidt C, Sciacovelli M, Frezza C (2020). Fumarate hydratase in cancer: A multifaceted tumour suppressor. Seminars in Cell & Developmental Biology.

[9] Lloyd MC, Cunningham JJ, Bui MM, Gillies RJ, Brown JS, Gatenby RA (2016). Darwinian dynamics of intratumoral heterogeneity: Not solely random mutations but also variable environmental selection forces. Cancer Research.

[10] Gillies RJ, Brown JS, Anderson ARA, Gatenby RA (2018). Eco-evolutionary causes and consequences of temporal changes in intratumoural blood flow. Nature Reviews Cancer.

[11] Elia I, Haigis MC (2021). Metabolites and the tumour microenvironment: From cellular mechanisms to systemic metabolism. Nature Metabolism.

[12] Harris AL (2002). Hypoxia–A key regulatory factor in tumour growth. Nature Reviews Cancer.

[13] Ratcliffe PJ (2013). Oxygen sensing and hypoxia signalling pathways in animals: The implications of physiology for cancer. Journal of Physiology.

[14] Semenza GL (2012). Hypoxia-inducible factors: Mediators of cancer progression and targets for cancer therapy. Trends in Pharmacological Sciences.

[15] Wouters BG, Koritzinsky M (2008). Hypoxia signalling through mTOR and the unfolded protein response in cancer. Nature Reviews Cancer.

[16] Sebestyen A, Kopper L, Danko T, Timar J (2021). Hypoxia signaling in cancer: From basics to clinical practice. Pathology Oncology Research.

[17] Li F, Simon MC (2020). Cancer cells don’t live alone: Metabolic communication within tumor microenvironments. Developmental Cell.

[18] Corbet C, Feron O (2017). Tumour acidosis: From the passenger to the driver's seat. Nature Reviews Cancer.

[19] Sonveaux P, Vegran F, Schroeder T, Wergin MC, Verrax J, Rabbani ZN (2008). Targeting lactate-fueled respiration selectively kills hypoxic tumor cells in mice. The Journal of Clinical Investigation.

[20] Payen VL, Mina E, Van Hee VF, Porporato PE, Sonveaux P (2020). Monocarboxylate transporters in cancer. Mol Metab.

[21] Swietach P (2019). What is pH regulation, and why do cancer cells need it?. Cancer and Metastasis Reviews.

[22] Wojtkowiak JW, Verduzco D, Schramm KJ, Gillies RJ (2011). Drug resistance and cellular adaptation to tumor acidic pH microenvironment. Molecular Pharmaceutics.

[23] Schonichen A, Webb BA, Jacobson MP, Barber DL (2013). Considering protonation as a posttranslational modification regulating protein structure and function. Annual Review of Biophysics.

[24] Dixon SJ, Lemberg KM, Lamprecht MR, Skouta R, Zaitsev EM, Gleason CE (2012). Ferroptosis: An iron-dependent form of nonapoptotic cell death. Cell.

[25] Chafe, S. C., Vizeacoumar, F. S., Venkateswaran, G., Nemirovsky, O., Awrey, S., Brown, W. S., et al. (2021). Genome-wide synthetic lethal screen unveils novel CAIX-NFS1/xCT axis as a targetable vulnerability in hypoxic solid tumors. *Sci Adv, 7*(35). 10.1126/sciadv.abj0364. 10.1126/sciadv.abj0364 PMC8397268 34452919

[26] Korenchan, D. E., & Flavell, R. R. (2019). Spatiotemporal pH heterogeneity as a promoter of cancer progression and therapeutic resistance. *Cancers (Basel), 11*(7). 10.3390/cancers11071026. 10.3390/cancers11071026 PMC6678451 31330859

[27] Mason JA, Hagel KR, Hawk MA, Schafer ZT (2017). Metabolism during ECM detachment: Achilles heel of cancer cells?. Trends Cancer.

[28] Sciacovelli M, Frezza C (2017). Metabolic reprogramming and epithelial-to-mesenchymal transition in cancer. FEBS Journal.

[29] Mosier JA, Schwager SC, Boyajian DA, Reinhart-King CA (2021). Cancer cell metabolic plasticity in migration and metastasis. Clinical & Experimental Metastasis.

[30] Chen Y, McAndrews KM, Kalluri R (2021). Clinical and therapeutic relevance of cancer-associated fibroblasts. Nature Reviews. Clinical Oncology.

[31] Fiaschi T, Marini A, Giannoni E, Taddei ML, Gandellini P, De Donatis A (2012). Reciprocal metabolic reprogramming through lactate shuttle coordinately influences tumor-stroma interplay. Cancer Research.

[32] Hulikova A, Swietach P (2014). Rapid CO2 permeation across biological membranes: Implications for CO2 venting from tissue. The FASEB Journal.

[33] Rohani N, Hao L, Alexis MS, Joughin BA, Krismer K, Moufarrej MN (2019). Acidification of tumor at stromal boundaries drives transcriptome alterations associated with aggressive phenotypes. Cancer Research.

[34] Chiche J, Brahimi-Horn MC, Pouyssegur J (2010). Tumour hypoxia induces a metabolic shift causing acidosis: A common feature in cancer. Journal of Cellular and Molecular Medicine.

[35] Reshkin SJ, Greco MR, Cardone RA (2014). Role of pHi, and proton transporters in oncogene-driven neoplastic transformation. Philosophical Transactions of the Royal Society of London. Series B, Biological sciences.

[36] Parks SK, Chiche J, Pouyssegur J (2011). pH control mechanisms of tumor survival and growth. Journal of Cellular Physiology.

[37] Fang JS, Gillies RD, Gatenby RA (2008). Adaptation to hypoxia and acidosis in carcinogenesis and tumor progression. Seminars in Cancer Biology.

[38] Gillies RJ (2021). Cancer heterogeneity and metastasis: Life at the edge. Clinical & Experimental Metastasis.

[39] Pastorek J, Pastorekova S, Callebaut I, Mornon JP, Zelnik V, Opavsky R (1994). Cloning and characterization of MN, a human tumor-associated protein with a domain homologous to carbonic anhydrase and a putative helix-loop-helix DNA binding segment. Oncogene.

[40] Opavsky R, Pastorekova S, Zelnik V, Gibadulinova A, Stanbridge EJ, Zavada J (1996). Human MN/CA9 gene, a novel member of the carbonic anhydrase family: Structure and exon to protein domain relationships. Genomics.

[41] Innocenti A, Pastorekova S, Pastorek J, Scozzafava A, De Simone G, Supuran CT (2009). The proteoglycan region of the tumor-associated carbonic anhydrase isoform IX acts as anintrinsic buffer optimizing CO2 hydration at acidic pH values characteristic of solid tumors. Bioorganic & Medicinal Chemistry Letters.

[42] Mahon BP, Bhatt A, Socorro L, Driscoll JM, Okoh C, Lomelino CL (2016). The structure of carbonic anhydrase IX is adapted for low-pH catalysis. Biochemistry.

[43] Wykoff CC, Beasley NJ, Watson PH, Turner KJ, Pastorek J, Sibtain A (2000). Hypoxia-inducible expression of tumor-associated carbonic anhydrases. Cancer Research.

[44] Ditte P, Dequiedt F, Svastova E, Hulikova A, Ohradanova-Repic A, Zatovicova M (2011). Phosphorylation of carbonic anhydrase IX controls its ability to mediate extracellular acidification in hypoxic tumors. Cancer Research.

[45] McDonald PC, Chafe SC, Brown WS, Saberi S, Swayampakula M, Venkateswaran G (2019). Regulation of pH by carbonic anhydrase 9 mediates survival of pancreatic cancer cells with activated KRAS in response to hypoxia. Gastroenterology.

[46] Takacova M, Holotnakova T, Barathova M, Pastorekova S, Kopacek J, Pastorek J (2010). Src induces expression of carbonic anhydrase IX via hypoxia-inducible factor 1. Oncology Reports.

[47] Takacova M, Bullova P, Simko V, Skvarkova L, Poturnajova M, Feketeova L (2014). Expression pattern of carbonic anhydrase IX in Medullary thyroid carcinoma supports a role for RET-mediated activation of the HIF pathway. American Journal of Pathology.

[48] Panisova E, Kery M, Sedlakova O, Brisson L, Debreova M, Sboarina M (2017). Lactate stimulates CA IX expression in normoxic cancer cells. Oncotarget.

[49] Kappler, M., Pabst, U., Weinholdt, C., Taubert, H., Rot, S., Kaune, T., et al. (2019). Causes and consequences of a glutamine induced normoxic HIF1 Activity for the tumor metabolism. *Int J Mol Sci, 20*(19). 10.3390/ijms20194742. 10.3390/ijms20194742 PMC6802203 31554283

[50] Svastova E, Hulikova A, Rafajova M, Zat'ovicova M, Gibadulinova A, Casini A (2004). Hypoxia activates the capacity of tumor-associated carbonic anhydrase IX to acidify extracellular pH. FEBS Letters.

[51] Swietach P, Patiar S, Supuran CT, Harris AL, Vaughan-Jones RD (2009). The role of carbonic anhydrase 9 in regulating extracellular and intracellular ph in three-dimensional tumor cell growths. Journal of Biological Chemistry.

[52] Lee SH, McIntyre D, Honess D, Hulikova A, Pacheco-Torres J, Cerdan S (2018). Carbonic anhydrase IX is a pH-stat that sets an acidic tumour extracellular pH in vivo. British Journal of Cancer.

[53] Morgan PE, Pastorekova S, Stuart-Tilley AK, Alper SL, Casey JR (2007). Interactions of transmembrane carbonic anhydrase, CAIX, with bicarbonate transporters. American Journal of Physiology. Cell Physiology.

[54] Svastova E, Witarski W, Csaderova L, Kosik I, Skvarkova L, Hulikova A (2012). Carbonic anhydrase IX interacts with bicarbonate transporters in lamellipodia and increases cell migration via its catalytic domain. Journal of Biological Chemistry.

[55] Orlowski A, De Giusti VC, Morgan PE, Aiello EA, Alvarez BV (2012). Binding of carbonic anhydrase IX to extracellular loop 4 of the NBCe1 Na+/HCO3- cotransporter enhances NBCe1-mediated HCO3- influx in the rat heart. American Journal of Physiology. Cell Physiology.

[56] Jamali S, Klier M, Ames S, Barros LF, McKenna R, Deitmer JW (2015). Hypoxia-induced carbonic anhydrase IX facilitates lactate flux in human breast cancer cells by non-catalytic function. Science and Reports.

[57] Ames S, Pastorekova S, Becker HM (2018). The proteoglycan-like domain of carbonic anhydrase IX mediates non-catalytic facilitation of lactate transport in cancer cells. Oncotarget.

[58] Ames S, Andring JT, McKenna R, Becker HM (2019). CAIX forms a transport metabolon with monocarboxylate transporters in human breast cancer cells. Oncogene.

[59] Liskova, V., Hudecova, S., Lencesova, L., Iuliano, F., Sirova, M., Ondrias, K., et al. (2019). Type 1 sodium calcium exchanger forms a complex with carbonic anhydrase IX and via reverse mode activity contributes to pH control in hypoxic tumors. *Cancers (Basel), 11*(8). 10.3390/cancers11081139. 10.3390/cancers11081139 PMC6721473 31395807

[60] Chafe SC, McDonald PC, Saberi S, Nemirovsky O, Venkateswaran G, Burugu S (2019). Targeting hypoxia-induced carbonic anhydrase IX enhances immune-checkpoint blockade locally and systemically. Cancer Immunology Research.

[61] Chiche J, Ilc K, Laferriere J, Trottier E, Dayan F, Mazure NM (2009). Hypoxia-inducible carbonic anhydrase IX and XII promote tumor cell growth by counteracting acidosis through the regulation of the intracellular pH. Cancer Research.

[62] McIntyre A, Hulikova A, Ledaki I, Snell C, Singleton D, Steers G (2016). Disrupting hypoxia-induced bicarbonate transport acidifies tumor cells and suppresses tumor growth. Cancer Research.

[63] Parks SK, Cormerais Y, Durivault J, Pouyssegur J (2017). Genetic disruption of the pHi-regulating proteins Na+/H+ exchanger 1 (SLC9A1) and carbonic anhydrase 9 severely reduces growth of colon cancer cells. Oncotarget.

[64] Pacchiano F, Carta F, McDonald PC, Lou Y, Vullo D, Scozzafava A (2011). Ureido-substituted benzenesulfonamides potently inhibit carbonic anhydrase IX and show antimetastatic activity in a model of breast cancer metastasis. Journal of Medicinal Chemistry.

[65] Lock FE, McDonald PC, Lou Y, Serrano I, Chafe SC, Ostlund C (2013). Targeting carbonic anhydrase IX depletes breast cancer stem cells within the hypoxic niche. Oncogene.

[66] Ledaki I, McIntyre A, Wigfield S, Buffa F, McGowan S, Baban D (2015). Carbonic anhydrase IX induction defines a heterogeneous cancer cell response to hypoxia and mediates stem cell-like properties and sensitivity to HDAC inhibition. Oncotarget.

[67] Neri D, Supuran CT (2011). Interfering with pH regulation in tumours as a therapeutic strategy. Nature Reviews Drug Discovery.

[68] Pastorek J, Pastorekova S (2015). Hypoxia-induced carbonic anhydrase IX as a target for cancer therapy: From biology to clinical use. Seminars in Cancer Biology.

[69] Strapcova, S., Takacova, M., Csaderova, L., Martinelli, P., Lukacikova, L., Gal, V., et al. (2020). Clinical and pre-clinical evidence of carbonic anhydrase IX in pancreatic cancer and its high expression in pre-cancerous lesions. *Cancers (Basel), 12*(8). 10.3390/cancers12082005. 10.3390/cancers12082005 PMC7464147 32707920

[70] Kery M, Oravcova N, Radenkovic S, Iuliano F, Tomaskova J, Golias T (2018). Pyruvate dehydrogenase kinase 1 and carbonic anhydrase IX targeting in hypoxic tumors. Neoplasma.

[71] Gibadulinova A, Bullova P, Strnad H, Pohlodek K, Jurkovicova D, Takacova M (2020). CAIX-mediated control of LIN28/let-7 axis contributes to metabolic adaptation of breast cancer cells to hypoxia. International Journal of Molecular Sciences.

[72] Benej M, Svastova E, Banova R, Kopacek J, Gibadulinova A, Kery M (2020). CA IX stabilizes intracellular pH to maintain metabolic reprogramming and proliferation in hypoxia. Frontiers in Oncology.

[73] Becker HM (2020). Carbonic anhydrase IX and acid transport in cancer. British Journal of Cancer.

[74] Harguindey S, Arranz JL, Polo Orozco JD, Rauch C, Fais S, Cardone RA (2013). Cariporide and other new and powerful NHE1 inhibitors as potentially selective anticancer drugs–An integral molecular/biochemical/metabolic/clinical approach after one hundred years of cancer research. Journal of Translational Medicine.

[75] Lamonte G, Tang X, Chen JL, Wu J, Ding CK, Keenan MM (2013). Acidosis induces reprogramming of cellular metabolism to mitigate oxidative stress. Cancer Metab.

[76] Svastova E, Pastorekova S (2013). Carbonic anhydrase IX: A hypoxia-controlled “catalyst” of cell migration. Cell Adhesion & Migration.

[77] McDonald, P. C., Swayampakula, M., & Dedhar, S. (2018). Coordinated regulation of metabolic transporters and migration/invasion by carbonic anhydrase IX. *Metabolites, 8*(1). 10.3390/metabo8010020. 10.3390/metabo8010020 PMC5876009 29517989

[78] Csaderova L, Debreova M, Radvak P, Stano M, Vrestiakova M, Kopacek J (2013). The effect of carbonic anhydrase IX on focal contacts during cell spreading and migration. Frontiers in Physiology.

[79] Radvak P, Repic M, Svastova E, Takacova M, Csaderova L, Strnad H (2013). Suppression of carbonic anhydrase IX leads to aberrant focal adhesion and decreased invasion of tumor cells. Oncology Reports.

[80] Svastova E, Zilka N, Zat'ovicova M, Gibadulinova A, Ciampor F, Pastorek J (2003). Carbonic anhydrase IX reduces E-cadherin-mediated adhesion of MDCK cells via interaction with beta-catenin. Experimental Cell Research.

[81] Debreova, M., Csaderova, L., Burikova, M., Lukacikova, L., Kajanova, I., Sedlakova, O., et al. (2019). CAIX regulates invadopodia formation through both a pH-dependent mechanism and interplay with actin regulatory proteins. *Int J Mol Sci, 20*(11). 10.3390/ijms20112745. 10.3390/ijms20112745 PMC6600150 31167468

[82] Swayampakula M, McDonald PC, Vallejo M, Coyaud E, Chafe SC, Westerback A (2017). The interactome of metabolic enzyme carbonic anhydrase IX reveals novel roles in tumor cell migration and invadopodia/MMP14-mediated invasion. Oncogene.

[83] Venkateswaran G, Dedhar S (2020). Interplay of carbonic anhydrase IX with amino acid and acid/base transporters in the hypoxic tumor microenvironment. Front Cell Dev Biol.

[84] Stock C, Schwab A (2009). Protons make tumor cells move like clockwork. Pflugers Archiv. European Journal of Physiology.

[85] LeBleu, V. S., O'Connell, J. T., Gonzalez Herrera, K. N., Wikman, H., Pantel, K., Haigis, M. C., et al. (2014). PGC-1alpha mediates mitochondrial biogenesis and oxidative phosphorylation in cancer cells to promote metastasis. *Nat Cell Biol, 16*(10), 992–1003, 1001–1015. 10.1038/ncb3039. 10.1038/ncb3039 PMC4369153 25241037

[86] Jiang L, Xiao L, Sugiura H, Huang X, Ali A, Kuro-o M (2015). Metabolic reprogramming during TGFbeta1-induced epithelial-to-mesenchymal transition. Oncogene.

[87] Hui EP, Chan AT, Pezzella F, Turley H, To KF, Poon TC (2002). Coexpression of hypoxia-inducible factors 1alpha and 2alpha, carbonic anhydrase IX, and vascular endothelial growth factor in nasopharyngeal carcinoma and relationship to survival. Clinical Cancer Research.

[88] Tomes L, Emberley E, Niu Y, Troup S, Pastorek J, Strange K (2003). Necrosis and hypoxia in invasive breast carcinoma. Breast Cancer Research and Treatment.

[89] Rademakers SE, Lok J, van der Kogel AJ, Bussink J, Kaanders JH (2011). Metabolic markers in relation to hypoxia; Staining patterns and colocalization of pimonidazole, HIF-1alpha, CAIX, LDH-5, GLUT-1, MCT1 and MCT4. BMC Cancer.

[90] Mayer A, Schneider F, Vaupel P, Sommer C, Schmidberger H (2012). Differential expression of HIF-1 in glioblastoma multiforme and anaplastic astrocytoma. International Journal of Oncology.

[91] Rohan SM, Xiao Y, Liang Y, Dudas ME, Al-Ahmadie HA, Fine SW (2011). Clear-cell papillary renal cell carcinoma: Molecular and immunohistochemical analysis with emphasis on the von Hippel-Lindau gene and hypoxia-inducible factor pathway-related proteins. Modern Pathology.

[92] Ord JJ, Streeter EH, Roberts IS, Cranston D, Harris AL (2005). Comparison of hypoxia transcriptome in vitro with in vivo gene expression in human bladder cancer. British Journal of Cancer.

[93] Airley RE, Loncaster J, Raleigh JA, Harris AL, Davidson SE, Hunter RD (2003). GLUT-1 and CAIX as intrinsic markers of hypoxia in carcinoma of the cervix: Relationship to pimonidazole binding. International Journal of Cancer.

[94] Dooms C, van Baardwijk A, Verbeken E, van Suylen RJ, Stroobants S, De Ruysscher D (2009). Association between 18F-fluoro-2-deoxy-D-glucose uptake values and tumor vitality: Prognostic value of positron emission tomography in early-stage non-small cell lung cancer. Journal of Thoracic Oncology.

[95] Koukourakis MI, Pitiakoudis M, Giatromanolaki A, Tsarouha A, Polychronidis A, Sivridis E (2006). Oxygen and glucose consumption in gastrointestinal adenocarcinomas: Correlation with markers of hypoxia, acidity and anaerobic glycolysis. Cancer Science.

[96] Schmidt DR, Patel R, Kirsch DG, Lewis CA, Vander Heiden MG, Locasale JW (2021). Metabolomics in cancer research and emerging applications in clinical oncology. CA: A Cancer Journal for Clinicians.

[97] Muir, A., Danai, L. V., & Vander Heiden, M. G. (2018). Microenvironmental regulation of cancer cell metabolism: implications for experimental design and translational studies. *Dis Model Mech, 11*(8). 10.1242/dmm.035758. 10.1242/dmm.035758 PMC6124553 30104199

[98] Bjorling E, Uhlen M (2008). Antibodypedia, a portal for sharing antibody and antigen validation data. Molecular and Cellular Proteomics.

[99] Helsby MA, Leader PM, Fenn JR, Gulsen T, Bryant C, Doughton G (2014). CiteAb: A searchable antibody database that ranks antibodies by the number of times they have been cited. BMC Cell Biology.

[100] van Kuijk SJ, Yaromina A, Houben R, Niemans R, Lambin P, Dubois LJ (2016). Prognostic significance of carbonic anhydrase IX expression in cancer patients: A meta-analysis. Frontiers in Oncology.

[101] Weller MG (2018). Ten Basic Rules of Antibody Validation. Analytical Chemistry Insights.

[102] Dungwa JV, Hunt LP, Ramani P (2012). Carbonic anhydrase IX up-regulation is associated with adverse clinicopathologic and biologic factors in neuroblastomas. Human Pathology.

[103] Korkolopoulou P, Perdiki M, Thymara I, Boviatsis E, Agrogiannis G, Kotsiakis X (2007). Expression of hypoxia-related tissue factors in astrocytic gliomas. A multivariate survival study with emphasis upon carbonic anhydrase IX. Hum Pathol.

[104] Ameis HM, Drenckhan A, Freytag M, Izbicki JR, Supuran CT, Reinshagen K (2016). Carbonic anhydrase IX correlates with survival and is a potential therapeutic target for neuroblastoma. Journal of Enzyme Inhibition and Medicinal Chemistry.

[105] Jarvela S, Parkkila S, Bragge H, Kahkonen M, Parkkila AK, Soini Y (2008). Carbonic anhydrase IX in oligodendroglial brain tumors. BMC Cancer.

[106] Nordfors K, Haapasalo J, Korja M, Niemela A, Laine J, Parkkila AK (2010). The tumour-associated carbonic anhydrases CA II, CA IX and CA XII in a group of medulloblastomas and supratentorial primitive neuroectodermal tumours: An association of CA IX with poor prognosis. BMC Cancer.

[107] Haapasalo JA, Nordfors KM, Hilvo M, Rantala IJ, Soini Y, Parkkila AK (2006). Expression of carbonic anhydrase IX in astrocytic tumors predicts poor prognosis. Clinical Cancer Research.

[108] Erpolat OP, Gocun PU, Akmansu M, Ozgun G, Akyol G (2013). Hypoxia-related molecules HIF-1alpha, CA9, and osteopontin: Predictors of survival in patients with high-grade glioma. Strahlentherapie und Onkologie.

[109] Proescholdt MA, Merrill MJ, Stoerr EM, Lohmeier A, Pohl F, Brawanski A (2012). Function of carbonic anhydrase IX in glioblastoma multiforme. Neuro-Oncology.

[110] Yoo H, Sohn S, Nam BH, Min HS, Jung E, Shin SH (2010). The expressions of carbonic anhydrase 9 and vascular endothelial growth factor in astrocytic tumors predict a poor prognosis. International Journal of Molecular Medicine.

[111] Abraham S, Hu N, Jensen R (2012). Hypoxia-inducible factor-1-regulated protein expression and oligodendroglioma patient outcome: Comparison with established biomarkers and preoperative UCSF low-grade scoring system. Journal of Neuro-oncology.

[112] Jensen R, Lee J (2012). Predicting outcomes of patients with intracranial meningiomas using molecular markers of hypoxia, vascularity, and proliferation. Neurosurgery.

[113] Sooman L, Freyhult E, Jaiswal A, Navani S, Edqvist PH, Ponten F (2015). FGF2 as a potential prognostic biomarker for proneural glioma patients. Acta Oncologica.

[114] Flynn JR, Wang L, Gillespie DL, Stoddard GJ, Reid JK, Owens J (2008). Hypoxia-regulated protein expression, patient characteristics, and preoperative imaging as predictors of survival in adults with glioblastoma multiforme. Cancer.

[115] Preusser M, Wolfsberger S, Haberler C, Breitschopf H, Czech T, Slavc I (2005). Vascularization and expression of hypoxia-related tissue factors in intracranial ependymoma and their impact on patient survival. Acta Neuropathologica.

[116] Couvelard A, O'Toole D, Turley H, Leek R, Sauvanet A, Degott C (2005). Microvascular density and hypoxia-inducible factor pathway in pancreatic endocrine tumours: Negative correlation of microvascular density and VEGF expression with tumour progression. British Journal of Cancer.

[117] Couvelard A, O'Toole D, Leek R, Turley H, Sauvanet A, Degott C (2005). Expression of hypoxia-inducible factors is correlated with the presence of a fibrotic focus and angiogenesis in pancreatic ductal adenocarcinomas. Histopathology.

[118] Chang DT, Chapman CH, Norton JA, Visser B, Fisher GA, Kunz P (2010). Expression of p16(INK4A) but not hypoxia markers or poly adenosine diphosphate-ribose polymerase is associated with improved survival in patients with pancreatic adenocarcinoma. Cancer.

[119] Hiraoka N, Ino Y, Sekine S, Tsuda H, Shimada K, Kosuge T (2010). Tumour necrosis is a postoperative prognostic marker for pancreatic cancer patients with a high interobserver reproducibility in histological evaluation. British Journal of Cancer.

[120] Li Y, Dong M, Sheng W, Huang L (2016). Roles of carbonic anhydrase IX in development of pancreatic cancer. Pathology Oncology Research.

[121] Schmitt AM, Schmid S, Rudolph T, Anlauf M, Prinz C, Kloppel G (2009). VHL inactivation is an important pathway for the development of malignant sporadic pancreatic endocrine tumors. Endocrine-Related Cancer.

[122] Yu M, Zhou Q, Zhou Y, Fu Z, Tan L, Ye X (2015). Metabolic phenotypes in pancreatic cancer. PLoS ONE.

[123] Trastour C, Benizri E, Ettore F, Ramaioli A, Chamorey E, Pouyssegur J (2007). HIF-1alpha and CA IX staining in invasive breast carcinomas: Prognosis and treatment outcome. International Journal of Cancer.

[124] Hussain SA, Ganesan R, Reynolds G, Gross L, Stevens A, Pastorek J (2007). Hypoxia-regulated carbonic anhydrase IX expression is associated with poor survival in patients with invasive breast cancer. British Journal of Cancer.

[125] Betof AS, Rabbani ZN, Hardee ME, Kim SJ, Broadwater G, Bentley RC (2012). Carbonic anhydrase IX is a predictive marker of doxorubicin resistance in early-stage breast cancer independent of HER2 and TOP2A amplification. British Journal of Cancer.

[126] Aomatsu N, Yashiro M, Kashiwagi S, Kawajiri H, Takashima T, Ohsawa M (2014). Carbonic anhydrase 9 is associated with chemosensitivity and prognosis in breast cancer patients treated with taxane and anthracycline. BMC Cancer.

[127] Lou Y, McDonald PC, Oloumi A, Chia S, Ostlund C, Ahmadi A (2011). Targeting tumor hypoxia: Suppression of breast tumor growth and metastasis by novel carbonic anhydrase IX inhibitors. Cancer Research.

[128] Tan EY, Yan M, Campo L, Han C, Takano E, Turley H (2009). The key hypoxia regulated gene CAIX is upregulated in basal-like breast tumours and is associated with resistance to chemotherapy. British Journal of Cancer.

[129] Brennan DJ, Jirstrom K, Kronblad A, Millikan RC, Landberg G, Duffy MJ (2006). CA IX is an independent prognostic marker in premenopausal breast cancer patients with one to three positive lymph nodes and a putative marker of radiation resistance. Clinical Cancer Research.

[130] Generali D, Fox SB, Berruti A, Brizzi MP, Campo L, Bonardi S (2006). Role of carbonic anhydrase IX expression in prediction of the efficacy and outcome of primary epirubicin/tamoxifen therapy for breast cancer. Endocrine-Related Cancer.

[131] Kyndi M, Sorensen FB, Knudsen H, Alsner J, Overgaard M, Nielsen HM (2008). Carbonic anhydrase IX and response to postmastectomy radiotherapy in high-risk breast cancer: A subgroup analysis of the DBCG82 b and c trials. Breast Cancer Research.

[132] Neumeister VM, Sullivan CA, Lindner R, Lezon-Geyda K, Li J, Zavada J (2012). Hypoxia-induced protein CAIX is associated with somatic loss of BRCA1 protein and pathway activity in triple negative breast cancer. Breast Cancer Research and Treatment.

[133] Doyen J, Trastour C, Ettore F, Peyrottes I, Toussant N, Gal J (2014). Expression of the hypoxia-inducible monocarboxylate transporter MCT4 is increased in triple negative breast cancer and correlates independently with clinical outcome. Biochemical and Biophysical Research Communications.

[134] Pinheiro C, Sousa B, Albergaria A, Paredes J, Dufloth R, Vieira D (2011). GLUT1 and CAIX expression profiles in breast cancer correlate with adverse prognostic factors and MCT1 overexpression. Histology and Histopathology.

[135] Garcia S, Dales JP, Charafe-Jauffret E, Carpentier-Meunier S, Andrac-Meyer L, Jacquemier J (2007). Poor prognosis in breast carcinomas correlates with increased expression of targetable CD146 and c-Met and with proteomic basal-like phenotype. Human Pathology.

[136] Bane AL, Whelan TJ, Pond GR, Parpia S, Gohla G, Fyles AW (2014). Tumor factors predictive of response to hypofractionated radiotherapy in a randomized trial following breast conserving therapy. Annals of Oncology.

[137] Kim HM, Jung WH, Koo JS (2014). Site-specific metabolic phenotypes in metastatic breast cancer. Journal of Translational Medicine.

[138] Lancashire LJ, Powe DG, Reis-Filho JS, Rakha E, Lemetre C, Weigelt B (2010). A validated gene expression profile for detecting clinical outcome in breast cancer using artificial neural networks. Breast Cancer Research and Treatment.

[139] Kornegoor R, Verschuur-Maes AH, Buerger H, Hogenes MC, de Bruin PC, Oudejans JJ (2012). Fibrotic focus and hypoxia in male breast cancer. Modern Pathology.

[140] Noh S, Kim JY, Koo JS (2014). Metabolic differences in estrogen receptor-negative breast cancer based on androgen receptor status. Tumour Biology.

[141] Currie MJ, Beardsley BE, Harris GC, Gunningham SP, Dachs GU, Dijkstra B (2013). Immunohistochemical analysis of cancer stem cell markers in invasive breast carcinoma and associated ductal carcinoma in situ: Relationships with markers of tumor hypoxia and microvascularity. Human Pathology.

[142] Deb S, Johansson I, Byrne D, Nilsson C, kConFab I, Constable L (2014). Nuclear HIF1A expression is strongly prognostic in sporadic but not familial male breast cancer. Mod Pathol.

[143] Beketic-Oreskovic L, Ozretic P, Rabbani ZN, Jackson IL, Sarcevic B, Levanat S (2011). Prognostic significance of carbonic anhydrase IX (CA-IX), endoglin (CD105) and 8-hydroxy-2'-deoxyguanosine (8-OHdG) in breast cancer patients. Pathology Oncology Research.

[144] Kaya AO, Gunel N, Benekli M, Akyurek N, Buyukberber S, Tatli H (2012). Hypoxia inducible factor-1 alpha and carbonic anhydrase IX overexpression are associated with poor survival in breast cancer patients. Journal of B.U.ON..

[145] Silva P, Slevin NJ, Sloan P, Valentine H, Ryder D, Price P (2010). Use of multiple biological markers in radiotherapy-treated head and neck cancer. Journal of Laryngology and Otology.

[146] Koukourakis MI, Giatromanolaki A, Sivridis E, Simopoulos K, Pastorek J, Wykoff CC (2001). Hypoxia-regulated carbonic anhydrase-9 (CA9) relates to poor vascularization and resistance of squamous cell head and neck cancer to chemoradiotherapy. Clinical Cancer Research.

[147] Koukourakis MI, Bentzen SM, Giatromanolaki A, Wilson GD, Daley FM, Saunders MI (2006). Endogenous markers of two separate hypoxia response pathways (hypoxia inducible factor 2 alpha and carbonic anhydrase 9) are associated with radiotherapy failure in head and neck cancer patients recruited in the CHART randomized trial. Journal of Clinical Oncology.

[148] Bernstein JM, Andrews TD, Slevin NJ, West CM, Homer JJ (2015). Prognostic value of hypoxia-associated markers in advanced larynx and hypopharynx squamous cell carcinoma. The Laryngoscope.

[149] Choi SW, Kim JY, Park JY, Cha IH, Kim J, Lee S (2008). Expression of carbonic anhydrase IX is associated with postoperative recurrence and poor prognosis in surgically treated oral squamous cell carcinoma. Human Pathology.

[150] De Schutter H, Landuyt W, Verbeken E, Goethals L, Hermans R, Nuyts S (2005). The prognostic value of the hypoxia markers CA IX and GLUT 1 and the cytokines VEGF and IL 6 in head and neck squamous cell carcinoma treated by radiotherapy +/- chemotherapy. BMC Cancer.

[151] Douglas CM, Bernstein JM, Ormston VE, Hall RC, Merve A, Swindell R (2013). Lack of prognostic effect of carbonic anhydrase-9, hypoxia inducible factor-1alpha and bcl-2 in 286 patients with early squamous cell carcinoma of the glottic larynx treated with radiotherapy. Clinical Oncology (Royal College of Radiologists).

[152] Heo K, Kim YH, Sung HJ, Li HY, Yoo CW, Kim JY (2012). Hypoxia-induced up-regulation of apelin is associated with a poor prognosis in oral squamous cell carcinoma patients. Oral Oncology.

[153] Kim SJ, Shin HJ, Jung KY, Baek SK, Shin BK, Choi J (2007). Prognostic value of carbonic anhydrase IX and Ki-67 expression in squamous cell carcinoma of the tongue. Japanese Journal of Clinical Oncology.

[154] Perez-Sayans M, Suarez-Penaranda JM, Pilar GD, Supuran CT, Pastorekova S, Barros-Angueira F (2012). Expression of CA-IX is associated with advanced stage tumors and poor survival in oral squamous cell carcinoma patients. Journal of Oral Pathology and Medicine.

[155] Wachters JE, Schrijvers ML, Slagter-Menkema L, Mastik M, de Bock GH, Langendijk JA (2013). Prognostic significance of HIF-1a, CA-IX, and OPN in T1–T2 laryngeal carcinoma treated with radiotherapy. The Laryngoscope.

[156] Eriksen JG, Overgaard J, Danish H, Neck Cancer Study, G (2007). Lack of prognostic and predictive value of CA IX in radiotherapy of squamous cell carcinoma of the head and neck with known modifiable hypoxia: An evaluation of the DAHANCA 5 study. Radiother Oncol.

[157] Le QT, Shi G, Cao H, Nelson DW, Wang Y, Chen EY (2005). Galectin-1: A link between tumor hypoxia and tumor immune privilege. Journal of Clinical Oncology.

[158] Nordsmark M, Eriksen JG, Gebski V, Alsner J, Horsman MR, Overgaard J (2007). Differential risk assessments from five hypoxia specific assays: The basis for biologically adapted individualized radiotherapy in advanced head and neck cancer patients. Radiotherapy and Oncology.

[159] Winter SC, Shah KA, Han C, Campo L, Turley H, Leek R (2006). The relation between hypoxia-inducible factor (HIF)-1alpha and HIF-2alpha expression with anemia and outcome in surgically treated head and neck cancer. Cancer.

[160] Koukourakis MI, Giatromanolaki A, Danielidis V, Sivridis E (2008). Hypoxia inducible factor (HIf1alpha and HIF2alpha) and carbonic anhydrase 9 (CA9) expression and response of head-neck cancer to hypofractionated and accelerated radiotherapy. International Journal of Radiation Biology.

[161] Kondo Y, Yoshikawa K, Omura Y, Shinohara A, Kazaoka Y, Sano J (2011). Clinicopathological significance of carbonic anhydrase 9, glucose transporter-1, Ki-67 and p53 expression in oral squamous cell carcinoma. Oncology Reports.

[162] Brockton N, Dort J, Lau H, Hao D, Brar S, Klimowicz A (2011). High stromal carbonic anhydrase IX expression is associated with decreased survival in P16-negative head-and-neck tumors. International Journal of Radiation Oncology Biology Physics.

[163] Brockton NT, Klimowicz AC, Bose P, Petrillo SK, Konno M, Rudmik L (2012). High stromal carbonic anhydrase IX expression is associated with nodal metastasis and decreased survival in patients with surgically-treated oral cavity squamous cell carcinoma. Oral Oncology.

[164] Zheng G, Peng C, Jia X, Gu Y, Zhang Z, Deng Y (2015). ZEB1 transcriptionally regulated carbonic anhydrase 9 mediates the chemoresistance of tongue cancer via maintaining intracellular pH. Molecular Cancer.

[165] Sakata K, Someya M, Nagakura H, Nakata K, Oouchi A, Takagi M (2008). Brachytherapy for oral tongue cancer: An analysis of treatment results with various biological markers. Japanese Journal of Clinical Oncology.

[166] Chen Y, Li X, Wu S, Xu G, Zhou Y, Gong L (2014). Expression of HIF-1alpha and CAIX in nasopharyngeal carcinoma and their correlation with patients’ prognosis. Medical Oncology.

[167] Hwa JS, Kwon OJ, Park JJ, Woo SH, Kim JP, Ko GH (2015). The prognostic value of immunohistochemical markers for oral tongue squamous cell carcinoma. European Archives of Oto-Rhino-Laryngology.

[168] Kwon OJ, Park JJ, Ko GH, Seo JH, Jeong BK, Kang KM (2015). HIF-1alpha and CA-IX as predictors of locoregional control for determining the optimal treatment modality for early-stage laryngeal carcinoma. Head and Neck.

[169] Han MW, Lee HJ, Cho KJ, Kim JS, Roh JL, Choi SH (2012). Role of FDG-PET as a biological marker for predicting the hypoxic status of tongue cancer. Head and Neck.

[170] Roh JL, Cho KJ, Kwon GY, Choi SH, Nam SY, Kim SY (2008). Prognostic values of pathologic findings and hypoxia markers in 21 patients with salivary duct carcinoma. Journal of Surgical Oncology.

[171] Roh JL, Cho KJ, Kwon GY, Ryu CH, Chang HW, Choi SH (2009). The prognostic value of hypoxia markers in T2-staged oral tongue cancer. Oral Oncology.

[172] Yang JS, Lin CW, Chuang CY, Su SC, Lin SH, Yang SF (2015). Carbonic anhydrase IX overexpression regulates the migration and progression in oral squamous cell carcinoma. Tumour Biology.

[173] Eckert AW, Lautner MH, Schutze A, Bolte K, Bache M, Kappler M (2010). Co-expression of Hif1alpha and CAIX is associated with poor prognosis in oral squamous cell carcinoma patients. Journal of Oral Pathology and Medicine.

[174] Jonathan RA, Wijffels KI, Peeters W, de Wilde PC, Marres HA, Merkx MA (2006). The prognostic value of endogenous hypoxia-related markers for head and neck squamous cell carcinomas treated with ARCON. Radiotherapy and Oncology.

[175] Rademakers SE, Hoogsteen IJ, Rijken PF, Oosterwijk E, Terhaard CH, Doornaert PA (2013). Pattern of CAIX expression is prognostic for outcome and predicts response to ARCON in patients with laryngeal cancer treated in a phase III randomized trial. Radiotherapy and Oncology.

[176] Gilchrist KW, Gray R, Fowble B, Tormey DC, Taylor SG, t.  (1993). Tumor necrosis is a prognostic predictor for early recurrence and death in lymph node-positive breast cancer: A 10-year follow-up study of 728 Eastern Cooperative Oncology Group patients. Journal of Clinical Oncology.

[177] Ord JJ, Agrawal S, Thamboo TP, Roberts I, Campo L, Turley H (2007). An investigation into the prognostic significance of necrosis and hypoxia in high grade and invasive bladder cancer. Journal of Urology.

[178] Ramos-Vara JA, Miller MA (2014). When tissue antigens and antibodies get along: Revisiting the technical aspects of immunohistochemistry–The red, brown, and blue technique. Veterinary Pathology.

[179] Hartling L, Milne A, Hamm MP, Vandermeer B, Ansari M, Tsertsvadze A (2013). Testing the Newcastle Ottawa Scale showed low reliability between individual reviewers. Journal of Clinical Epidemiology.

