# Supplementary material for: Understanding metabolic alterations and heterogeneity in cancer progression through validated immunodetection of key molecular components: a case of carbonic anhydrase IX
Source: Cancer Metastasis Rev. 2022 Jan 26;40(4):1035–53. doi: 10.1007/s10555-021-10011-5 (PMC8825433; doi:10.1007/s10555-021-10011-5)
Supplement: Supplementary file 1 — Supplementary file1 (PDF 10594 KB) [file 10555_2021_10011_MOESM1_ESM.pdf]

SUPPLEMENTARY INFORMATION

Understanding metabolic alterations and heterogeneity in cancer progression through validated immunodetection of key molecular components: a case of carbonic anhydrase IX

Martina Takacova*, Ivana Kajanova*, Maria Kolarcikova, Jana Lapinova, Miriam Zatovicova, and #Silvia Pastorekova

Biomedical Research Center of the Slovak Academy of Sciences, Institute of Virology, Department of Tumor Biology, Dubravska cesta 9, 845 05 Bratislava, Slovak Republic

* These authors contributed equally to this work

# Correspondence: Silvia Pastorekova, Tel: +421-2-59302401, silvia.pastorekova@savba.sk,

ORCID 0000-0001-8203-405X

Supplementary Table S1
Anti-CA IX antibodies. Basic information on validated antibodies regarding host, antibody type, immunogen and key applications.

Name	Host	Antibody type	Immunogen/epitope	Key applications
**M75**	M	monoclonal	PG region	WB, FACS, IP, IF, IHC, ELISA
**Abcam ab15086**	R	polyclonal	synthetic peptide (aa 359-459)	WB, FACS, IHC
**Cell Signaling Technology D10C10**	R	monoclonal	synthetic peptide (around Asp361)	WB, IHC
**Invitrogen/ThermoFisher Scientific GT12**	M	monoclonal	linear repetitive epitope in the PG region	WB, FACS, IF, ICC, IHC, IP
**Merck 2D3**	M	monoclonal	purified recombinant fragment expressed in E. coli	WB, FACS, IHC
**Novus Biologicals NB100-417**	R	polyclonal	synthetic peptide from the C-terminal region (aa 400-459)	WB, FACS, IF, ICC, IHC, ELISA, ChIP, Gel Supershift,
**R&D Systems AF2188**	G	polyclonal	mouse myeloma cell line NS0 derived recombinant protein Pro59 - Asp414	WB, FACS, ICC, IHC, IP
**Santa Cruz Biotechnology H-11**	M	monoclonal	aa 41-160	WB, IP, IF, IHC, ELISA
**Sigma-Aldrich SAB1300310**	R	polyclonal	N-terminal region	WB, ELISA

M – mouse, R – rabbit, G – goat, WB – Wester blotting, FACS – fluorescence-activated cell sorting, IHC – immunohistochemistry, IF – immunofluorescence, ICC – immunocytochemistry, ELISA – enzyme-linked immunosorbent assay, IP – immunoprecipitation, ChIP – chromatin immunoprecipitation

Supplementary Table S2: Dilutions of all anti-CA IX antibodies selected on the basis of manufacturers' recommendation and authors' experience. Data are summarized according to the application.

Name/Application	WB	ELISA	FACS	IF	IHC
**M75**	1:3 (medium)	1,2,3,5 µg/ml	0.5 µg/10^6 cells	1 µg/ml	1:100
**Abcam ab15086**	1:1,000	1,2,3,5 µg/ml	1 µg/10^6 cells	1:100	1:1,000
**Cell Signaling Technology D10C10**	1:500	1,2,3,5 µg/ml	1:100	1:100	1:100
**Invitrogen/ThermoFisher Scientific GT12**	1:100	1,2,3,5 µg/ml	1:100	1:100	1:100
**Merck 2D3**	1:500	1,2,3,5 µg/ml	1:200	1:100	1:1,000
**Novus Biologicals NB100-417**	1:2,000	1,2,3,5 µg/ml	0.5 µg/10^6 cells	1:1,000	1:1,000
**R&D Systems AF2188**	1:200	1,2,3,5 µg/ml	0.25 µg/10^6 cells	1:40	10 µg/ml
**Santa Cruz Biotechnology H-11**	1:200	1,2,3,5 µg/ml	5 µg/10^6 cells	1:100	1:100
**Sigma-Aldrich SAB1300310**	1:250	1,2,3,5 µg/ml	1:50	1:100	1:100

Materials and Methods

Cell culture

Canine MDCK epithelial cells (ATCC CCL-34), as well as human tumor cell line SiHa (ATCC HTB-35) and C-33a (ATCC HTB-31) derived from cervical carcinoma were cultivated in DMEM supplemented with 10% FCS (BioWhittaker, Verviers, Belgium) in a humidified atmosphere with 5% CO_2 at 37°C. Hypoxic treatments were performed in an anaerobic workstation (Ruskin Technologies, Bridgend, UK) in 1% O_2, 5% CO_2, 10% H_2, and 83% N_2 at 37°C. The generation of CA IX-expressing C-33a and MDCK, as well as MDCK CA IX delPG cells was described previously [1].

Enzyme-linked immunosorbent assay

The expression of CA IX in protein extracts derived from either C-33a CA IX (CA IX-positive) or C-33a neo (CA IX-negative) cells was verified using direct ELISA. Protein concentration was determined using BCA protein assay reagents (Pierce, Rockford, IL, USA). Two different concentrations of proteins, 5 µg and 10 µg, were coated on the surface of microplate wells overnight at 37°C. The CA IX-specific reactivity of both protein concentrations was tested with an increasing amount of primary antibody (1, 2, 3 and 5 µg) for 2 h at RT. Peroxidase-labeled swine anti-mouse/rabbit/goat IgG (Sigma-Aldrich, USA) diluted 1:10,000 was used as the secondary antibody for 2 h at RT. Results are expressed as O.D. values of absorbance measured at 492 nm.

FACS

Stably transfected MDCK cells (MDCK CA IX and MDCK neo) were scraped into culture medium, centrifuged at low speed, washed twice with Versene solution and incubated with the anti-CA IX primary antibody for 1 h at 4°C. Following the centrifugation and washing with Versene, cells were incubated with the secondary anti-mouse/rabbit/goat Alexa Fluor® 488-conjugated antibody (Invitrogen, Carlsbad, California) diluted 1:1,000 in DMEM+10% FCS for 30 min at RT. Pelleted cells were again washed twice with Versene and finally analyzed using a Guava EasyCyte plus flow cytometer (Millipore, Darmstadt, Germany). Data were analyzed with Cytosoft 5.2 software using Guava Express Pro (Millipore, Darmstadt, Germany). Debris, cell doublets and clumps were excluded from analyses by scatter gating, and a total of 10,000 single cells were analyzed for each sample.
In case of two antibodies recognizing the C-terminal region of CA IX protein (2D3 and NB100-417), MDCK cells were firstly fixed in ice-cold ethanol for 1 h at -20°C and washed twice with PBS. Following the incubation with primary antibody, the staining procedure was similar as mentioned before.

Western blotting

SiHa (normoxic vs hypoxic), C-33a (neo vs CA IX) and MDCK (CA IX vs delPG) cells were rinsed twice with PBS, resuspended in ice-cold lysis buffer (0.1% deoxycholic acid, 1% Triton X-100 in PBS) containing a protease inhibitors cocktail (Roche, Basel, Switzerland) and cleared by centrifugation. Protein concentration was quantified using the BCA protein assay reagents (Pierce, Rockford, IL, USA). Total protein extracts were separated in 10% SDS-PAGE and transferred onto PVDF membrane (Macherey-Nagel, Düren, Germany). The incubation with anti-CA IX primary antibody was performed according to the manufacturers' recommendation for either 1 h at 37°C, or O/N at 4°C. Following the washing step, membranes were incubated with secondary anti-mouse/rabbit/goat-HRP antibody diluted 1:5,000. For a loading control, the membranes were probed with mouse monoclonal anti-actin antibody (Cell Signaling, Danvers, MA, USA). Protein bands were visualized using an enhanced chemiluminescence kit (GE Healthcare Bio-Sciences, Chicago, IL, USA).

Immunofluorescence

MDCK neo, MDCK CA IX and SiHa (pre-incubated 48 h in hypoxia) cells grown on glass coverslips were gently washed with PBS and fixed in ice-cold methanol at −20 °C for 5 min. Nonspecific binding was blocked by incubation with PBS containing 1% BSA for 30 min at 37°C. Cells were then incubated with anti-CA IX primary antibodies for 1 h at 37°C followed by a secondary (anti-mouse/rabbit/goat) Alexa Fluor® 488-conjugated antibody (Invitrogen, CA, USA) diluted 1:1,000 in the blocking buffer for 1 h at 37°C. The nuclei were stained with DAPI (Sigma-Aldrich, MO, USA). Finally, the coverslips were mounted onto slides in the Fluorescent Mounting Media (Sigma-Aldrich, MO, USA), and analyzed by the confocal laser scanning microscope Zeiss LSM 510 Meta.

Immunohistochemistry

Tissue samples derived from clear cell renal cell carcinoma (CCRCC) and healthy kidney were obtained from the Department of Urology, Derer's University Hospital Bratislava, Slovakia [2]. Before immunostaining, all slides were routinely deparaffinized and rehydrated. For each antibody, two different heat-induced epitope retrievals (citrate buffer, pH 6.0 and EDTA buffer, pH 9.0) were performed using Pascal pressure chamber (Dako) at 125°C for 5 min. Parallel set of slides was stained without antigen retrieval method. Primary antibody incubation time

and dilution were selected for each antibody on the basis of manufacturers' recommendation and authors' experience. The same detection system EnVision^{+}System-HRP (DAB) was used and all tissues were counterstained with Mayer hematoxylin for 5 min. In the negative controls, the primary antibodies were omitted. In serial sections stained with all antibodies, corresponding areas of the CCRCC were identified and examined. All sections were evaluated with regard to the distribution and intensity of the immunohistochemical reaction with a Leica DM4500B microscope and photographed with a Leica DFC480 camera.

Supplementary Figures

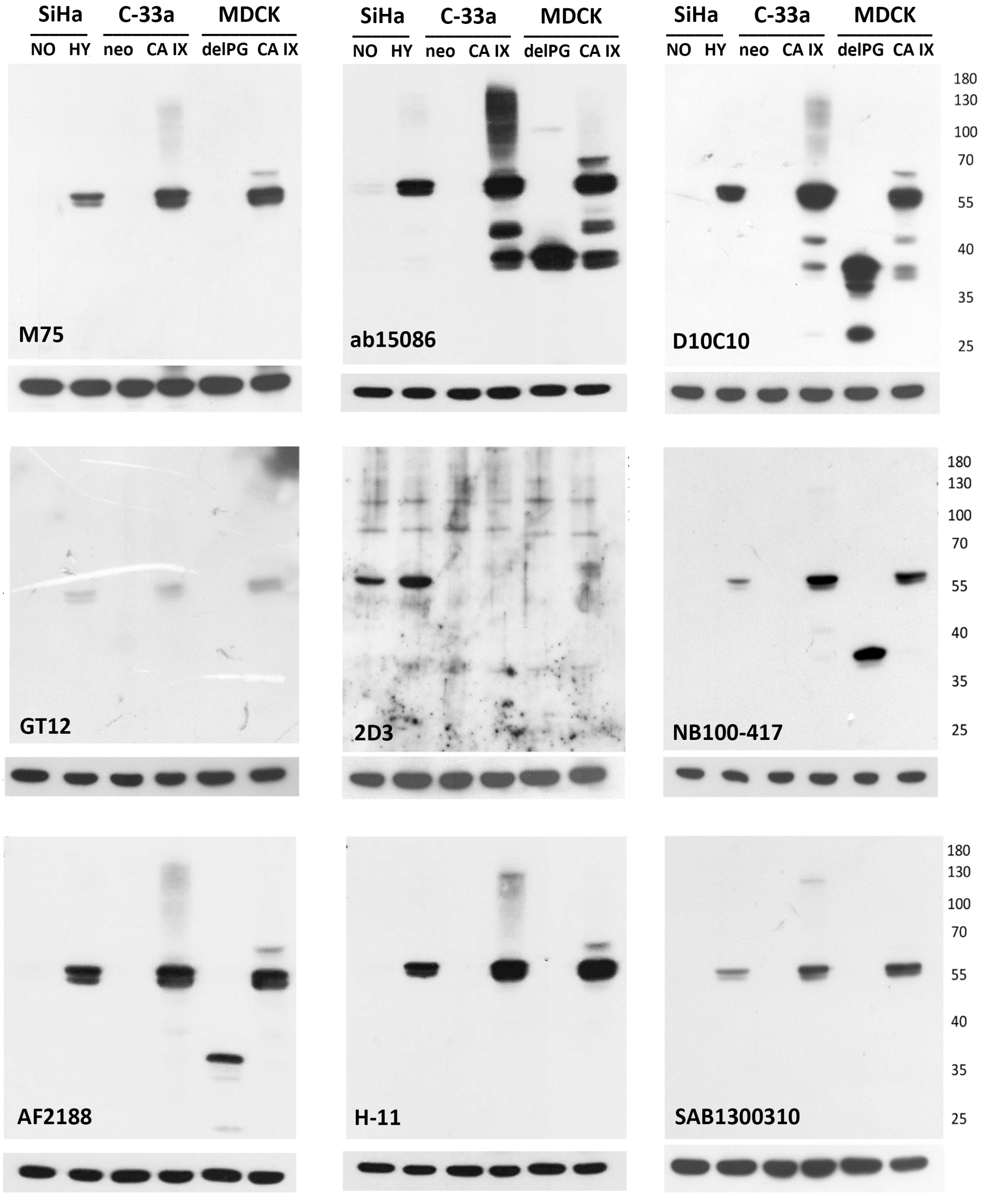

Supplementary Figure S1

Western blotting of representative cell lines expressing CA IX either naturally (SiHa after hypoxic induction) or ectopically (C-33a and MDCK after transfection). CA IX protein is observed as a typical twin band at 58 and 54 kDa in hypoxic SiHa cells (SiHa HY) as well as CA IX-transfected C-33a and MDCK cells (C-33a CA IX and MDCK CA IX). Mock transfected C-33a cells (C-33a neo) served as a negative control. As M75 is directed against N-terminal proteoglycan (PG) domain of CA IX protein, protein samples from MDCK cells expressing deletion variant delPG (MDCK delPG) were prepared and analyzed. Anti-actin antibody and Precision Plus Protein Standard was used for loading control and the molecular weight calibration, respectively.

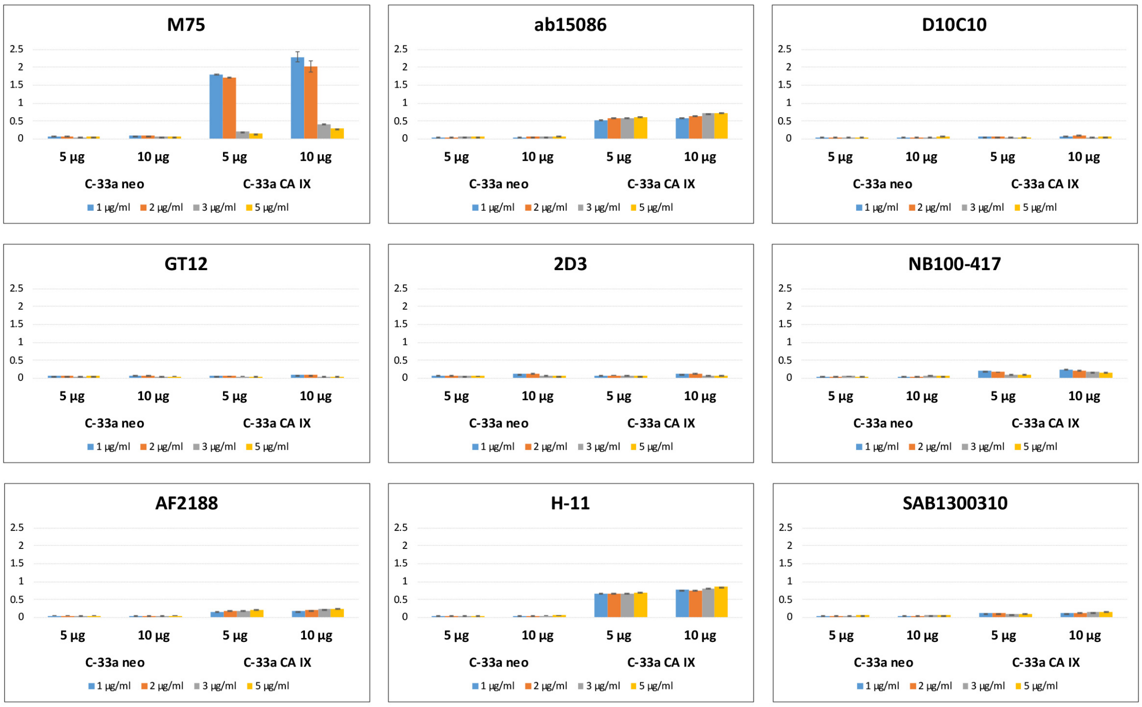

Supplementary Figure S2

Reactivity of M75 and eight selected antibodies in ELISA. Specificity of all tested antibodies was investigated using cell lysates from C-33a cells, CA IX-negative (C-33a neo) and CA IX-positive (C-33a CA IX). Increasing concentration of primary antibody (1, 2, 3 and 5 µg) as well as two different protein concentrations (5 µg vs 10 µg) of antigen were used. Results are expressed as O.D. values of absorbance measured at 492 nm and represent mean ± standard deviation values.

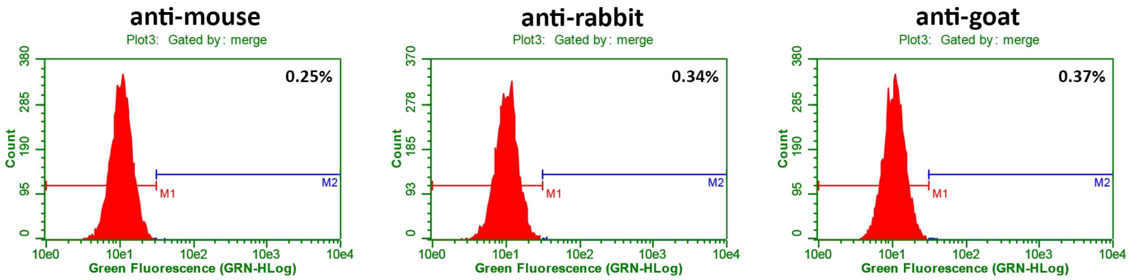

Supplementary Figure S3

Flow cytometry analysis of CA IX-expressing MDCK cells using only secondary anti-mouse/rabbit/goat antibody.

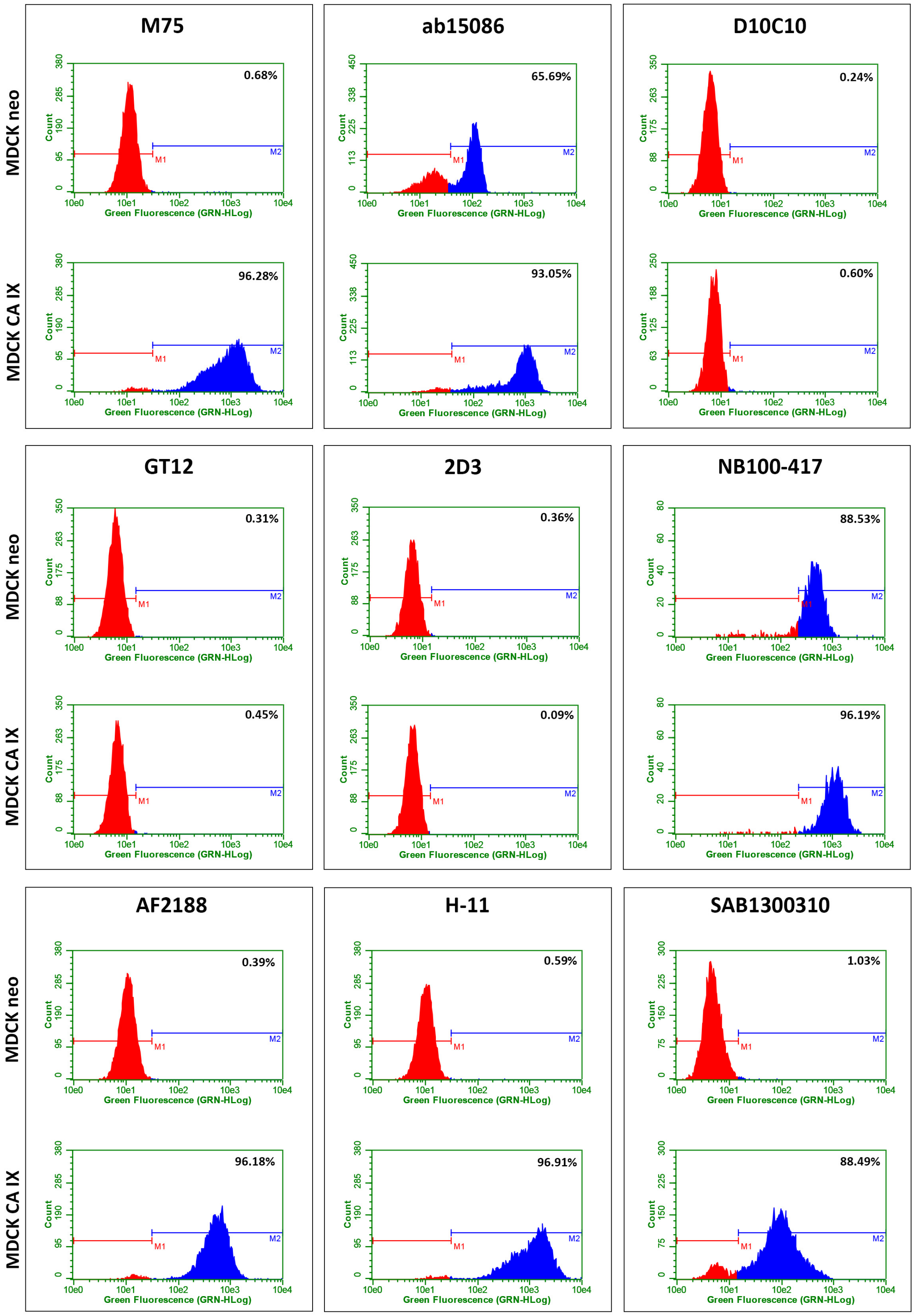

Supplementary Figure S4

Flow cytometry analysis comparing M75 with selected antibodies. Specificity of all tested antibodies was validated using both CA IX-negative (MDCK neo; upper graph) as well as CA IX-positive (MDCK CA IX; lower graph) MDCK cells. The percentage of CA IX-positive cells calculated from a total of 10,000 single cells are summarized on the right side of each graph.

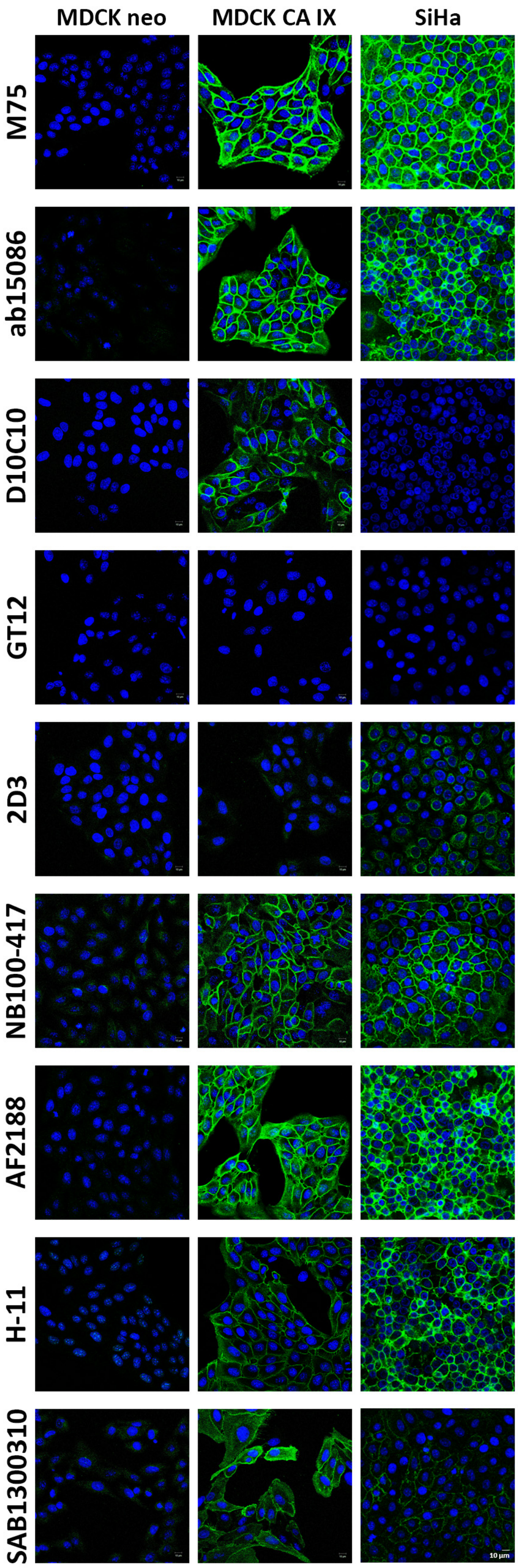

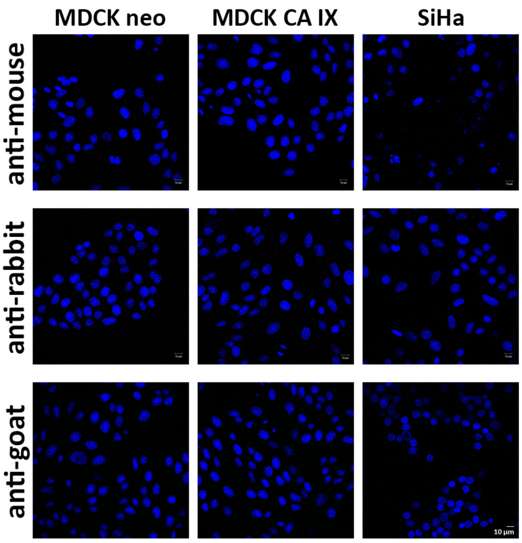

Supplementary Figure S5

Left: Immunofluorescence comparing M75 with eight commercial antibodies. Specificity of tested antibodies was validated using MDCK cells transfected with CA IX (MDCK CA IX) and SiHa cells pre-cultivated in hypoxia for 48 h. Mock transfected MDCK cell (MDCK neo) served as a negative control.

Right: Immunofluorescent analysis performed only with secondary antibodies. MDCK neo, MDCK CA IX and hypoxic SiHa cells were stained with Alexa Fluor® 488-conjugated anti-mouse/rabbit/goat secondary antibodies for 1 h at 37°C. The nuclei were stained with DAPI and analyzed by the confocal laser scanning microscope Zeiss LSM 510 Meta.

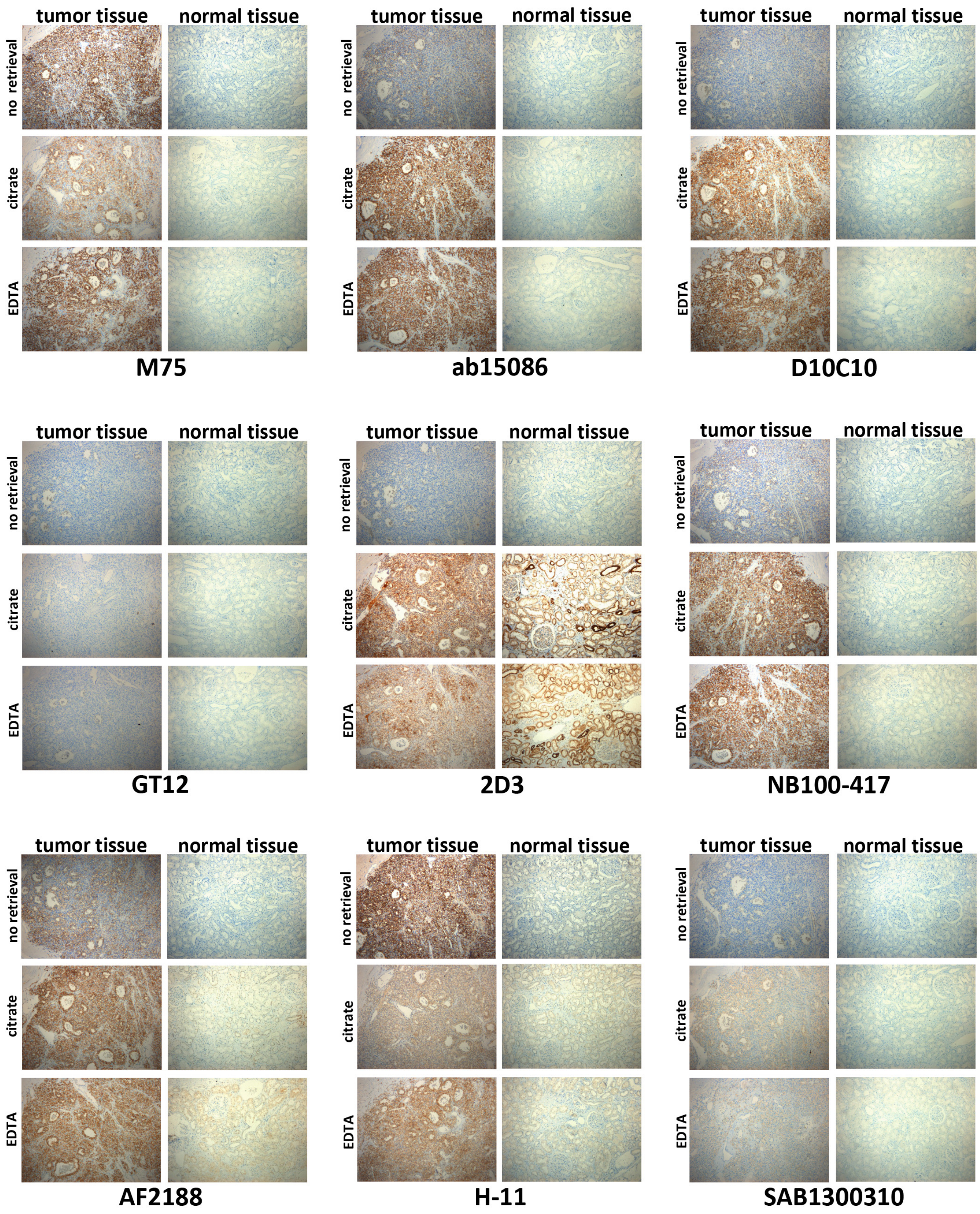

Supplementary Figure S6

Representative images from tumor and normal/healthy tissue stained using M75 and eight commercial antibodies. Serial tissue sections were either subjected to heat-induced antigen retrieval in citrate or EDTA buffer, or directly incubated in primary anti-CA IX antibody for 1 h at RT. Following the incubation with particular anti-mouse/rabbit/goat secondary antibody, positive reaction was visualized using DAB as a chromogen. All tissues were counterstained with Mayer hematoxylin and finally evaluated with regard to the distribution and intensity of the immunohistochemical reaction. High resolution images will be provided upon request.

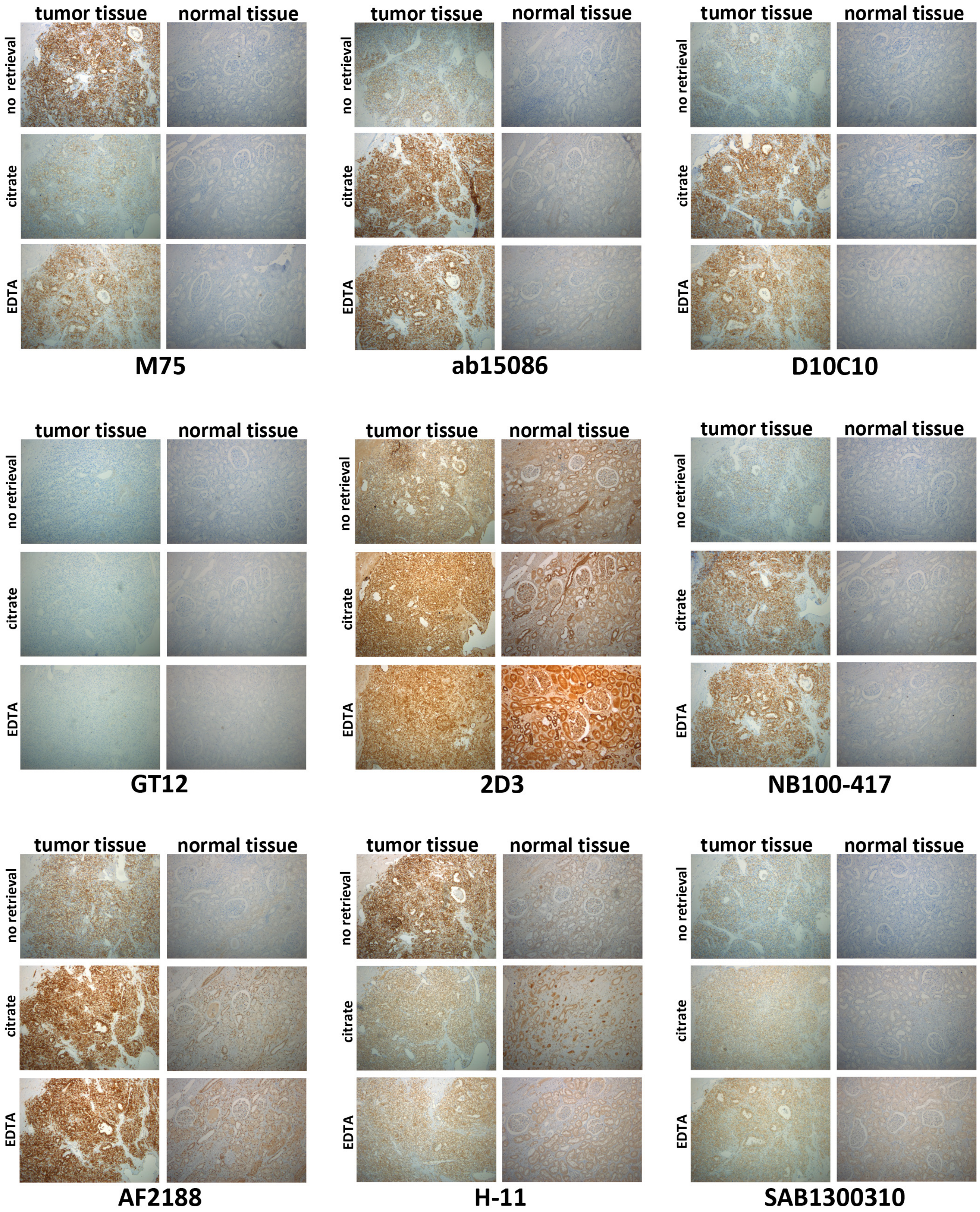

Supplementary Figure S6

Representative images from tumor and normal/healthy tissue stained using M75 and eight commercial antibodies. Serial tissue sections were either subjected to heat-induced antigen retrieval in citrate or EDTA buffer, or directly incubated in primary anti-CA IX antibody for 24 h at 4°C. Following the incubation with particular anti-mouse/rabbit/goat secondary antibody, positive reaction was visualized using DAB as a chromogen. All tissues were counterstained with Mayer hematoxylin and finally evaluated with regard to the distribution and intensity of the immunohistochemical reaction. High resolution images will be provided upon request.

References

1. Kajanova, I., Zatovicova, M., Jelenska, L., Sedlakova, O., Barathova, M., Csaderova, L., et al. (2020). Impairment of carbonic anhydrase IX ectodomain cleavage reinforces tumorigenic and metastatic phenotype of cancer cells. *Br J Cancer, 122*(11), 1590-1603, doi:10.1038/s41416-020-0804-z.
2. Takacova, M., Bartosova, M., Skvarkova, L., Zatovicova, M., Vidlickova, I., Csaderova, L., et al. (2013). Carbonic anhydrase IX is a clinically significant tissue and serum biomarker associated with renal cell carcinoma. *Oncol Lett, 5*(1), 191-197, doi:10.3892/ol.2012.1001.
